# Plasma GFAP and Amyloid Pathology Predict Cognitive Response to Multidomain Interventions in MCI

**DOI:** 10.14336/AD.2025.0646

**Published:** 2026-06-29

**Authors:** Myung-Hoon Han, Mina Hwang, Hyuk Sung Kwon, So Young Moon, Yoo Kyoung Park, Jee Hyang Jeong, Seong Hye Choi, Seong-Ho Koh

**Affiliations:** ^1^Department of Neurosurgery, Hanyang University Guri Hospital, Guri 11923, Republic of Korea.; ^2^Department of Neurology, Hanyang University Guri Hospital, Guri 11923, Republic of Korea.; ^3^Department of Neurology, Ajou University School of Medicine, Suwon 16499, Republic of Korea.; ^4^Department of Medical Nutrition, Graduate School of East-West Medical Nutrition, Kyung Hee University, Yongin 17104, Republic of Korea.; ^5^Department of Neurology, Ewha Womans University College of Medicine, Seoul 07804, Republic of Korea.; ^6^Department of Neurology, Inha University College of Medicine, Incheon 22332, Republic of Korea.; ^7^Department of Translational Medicine, Hanyang University Graduate School of Biomedical Science & Engineering, Seoul 04763, Republic of Korea

**Keywords:** GFAP, mild cognitive impairment (MCI), Alzheimer’s disease;, RBANS, amyloid beta deposition, multidomain intervention

## Abstract

There is limited evidence on which biological markers can predict the effectiveness of interventions in mild cognitive impairment (MCI) patients, particularly in relation to amyloid pathology. This study aims to investigate whether plasma glial fibrillary acidic protein (GFAP) levels can serve as a predictive biomarker for short-term cognitive response to multidomain interventions in elderly individuals with MCI, stratified by probable amyloid-β plaque deposition. In this 24-week multicenter randomized controlled trial (RCT; SUPERBRAIN-MEET), 300 elderly participants with MCI were enrolled. Probable amyloid status was determined using a plasma phosphorylated tau 181 cutoffs derived from a validated cohort. Multivariable linear regression analyses were employed to assess the association between plasma GFAP levels and percentage changes in Repeatable Battery for the Assessment of Neuropsychological Status (RBANS) scores, stratified by amyloid deposition status and intervention group. Higher plasma GFAP levels at baseline and 6 months were independently and significantly associated with smaller percentage improvements in RBANS scores over 6 months. Among participants with probable amyloid positivity who underwent the multidomain intervention, increased baseline GFAP levels were significantly associated with reduced cognitive improvement compared to those with lower levels (β = -8.661, p = 0.040). This post hoc exploratory subanalysis, based on data from a multicenter RCT, suggests that plasma GFAP may serve as a biomarker for early cognitive stage transitions in elderly individuals with MCI. Baseline GFAP levels—particularly among those with probable amyloid pathology—may help predict cognitive responsiveness to multidomain interventions.

## INTRODUCTION

Mild cognitive impairment (MCI) is a well-established intermediate stage between normal cognitive aging and dementia. Individuals diagnosed with MCI face an annual risk of progression to dementia of approximately 10-15%, emphasizing the importance of early identification of those at heightened risk [1]. Early detection enables timely interventions that may delay or mitigate disease progression.

Plasma biomarkers offer a promising, noninvasive method for detecting neurodegenerative processes and potentially underlying pathologies before the onset of clinical symptoms [2, 3]. Among these, glial fibrillary acidic protein (GFAP)—a cytoskeletal component of reactive astrocytes—has gained significant interest [2, 4]. Elevated plasma GFAP levels may reflect early neuroinflammatory responses linked to neuronal injury and are associated with subtle cognitive decline, even in prodromal stages of cognitive impairment [5]. Growing evidence suggests that amyloid pathology diminishes the efficacy of cognitive interventions, particularly in the context of astroglial activation. Elevated GFAP levels, reflecting reactive astrocytosis, may synergistically interact with amyloid deposition to exacerbate neuroinflammation and neuronal dysfunction, thereby attenuating cognitive recovery [6, 7]. Although prior studies have identified plasma GFAP as a potential biomarker for neurodegeneration and have explored the effects of lifestyle interventions in individuals with early AD, few have examined how baseline GFAP levels influence cognitive responsiveness to multidomain interventions, especially in the context of amyloid pathology. Furthermore, there is limited understanding of how astroglial activation, as measured by GFAP, may moderate the benefits of lifestyle-based cognitive interventions in MCI populations.

Recent studies have shown that plasma GFAP is not only a marker of reactive astrocytosis but also an indirect biomarker of cerebral amyloid-β pathology. Plasma GFAP levels correlate strongly with amyloid burden and often increase earlier than CSF GFAP, even before amyloid-PET positivity [8, 9]. Nonetheless, notable variability exists, with some Aβ-positive individuals showing low GFAP levels, possibly reflecting early or less-reactive astrocytic responses [9]. This heterogeneity may be important in understanding individual differences in response to cognitive interventions. This gap in knowledge hinders the development of tailored therapeutic strategies based on individual biomarker profiles.

A small randomized controlled trial (RCT) demonstrates that a multidomain lifestyle intervention, combined with nutritional supplementation, effectively improves cognitive outcomes in individuals with early symptomatic Alzheimer's disease (AD) [10]. Building on this, we recently conducted the SUPERBRAIN-MEET multicenter RCT, which shows that multidomain interventions targeting multiple modifiable risk factors significantly improve cognitive function, alleviate depressive symptoms, and enhance quality of life in individuals with MCI, thereby facilitating better management of the condition [11, 12].

To address the aforementioned gap, this study is a post hoc exploratory subanalysis of the SUPERBRAIN-MEET RCT, designed to investigate whether the cognitive benefits of a multidomain intervention differ based on the presence or absence of amyloid deposition. To infer probable amyloid status, we derived an optimal plasma phosphorylated tau 181 (pTau-181) cutoff using data from the Korean Brain Aging Study for the Early Diagnosis and Prediction of Alzheimer’s Disease-Validation (KBASE-V) cohort, where amyloid positivity was defined using Centiloid-calibrated amyloid positron emission tomography (PET imaging) [13-15]. This study aims to explore whether baseline plasma GFAP levels can serve as predictive biomarkers for short-term cognitive responsiveness to multidomain interventions in elderly individuals with MCI, particularly among those with probable amyloid pathology.

## MATERIALS AND METHODS

### Study design and participants

In this 24-week multicenter RCT, we used a two-parallel-group design with blinded outcome assessors. The experimental group underwent a multidomain intervention, while the control group served as a comparator. Older adults with MCI and memory concerns were recruited from outpatient clinics across 17 hospitals in Republic of Korea. The trial was registered on www.ClinicalTrials.gov platform (NCT05023057) [12].

Participants aged 60-85 years with MCI and at least one modifiable dementia risk factor, including hypertension, diabetes, dyslipidemia, obesity, hypothyroidism, depressive symptoms, or metabolic syndrome, were enrolled. The exclusion criteria included major depressive disorder, dementia, Parkinson’s disease, malignancy, or serious/unstable medical conditions. Participant selection and recruitment followed the criteria and procedures outlined in the SUPERBRAIN-MEET protocol [12].

**Figure 1.**
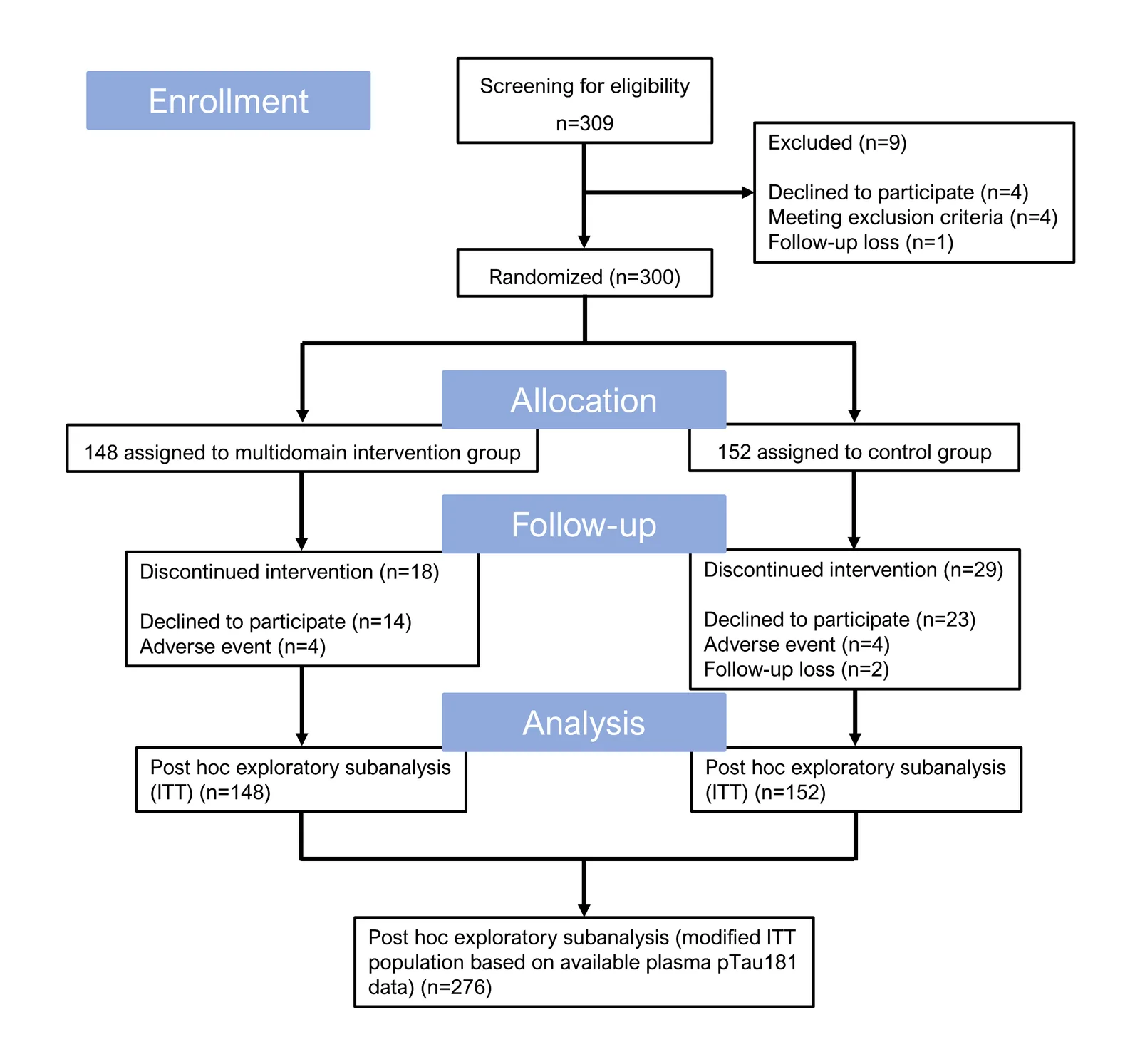
**Flow diagram of the RCT**. Abbreviations: ITT, intention-to-treat; pTau-181, phosphorylated tau 181; RCT, randomized controlled trial.

In addition to the primary findings previously reported, a post hoc exploratory subanalysis was conducted in this study using the same cohort [11]. These analyses primarily adhered to the intention-to-treat (ITT) principle to maintain randomization integrity and reduce bias in subgroup comparisons. For biomarker-based analyses involving plasma pTau-181 levels, a modified ITT (mITT) population was used, comprising participants with available biomarker data (Fig. 1).

### Randomization and intervention

Participants were randomly assigned in a 1:1 ratio to the intervention or control group using a permuted-block randomization method, implemented via SAS macro programming and stratified by the study center. Only an independent statistical specialist had access to the allocation sequence. Cognitive outcome assessors remained blinded to group assignments throughout the study.

The experimental group underwent a structured multidomain intervention comprising cognitive training, physical exercise, nutritional guidance, metabolic and vascular risk management, and motivational support. This intervention was delivered through a hybrid approach combining in-person and online sessions. Accessibility and engagement were facilitated using the SUPERBRAIN tablet application and Zoom platform. The experimental group underwent personalized feedback and ongoing monitoring of cognitive performance and vascular risk factors. Participants also engaged in self-directed activities at home and attended interactive group sessions to support adherence and motivation. This comprehensive approach targeted multiple modifiable risk factors and leveraged digital tools to enhance engagement and support scalability. The control group underwent standard care and a booklet with lifestyle guidelines for dementia prevention without active intervention. After the study, the control group participants were offered the opportunity to participate in the multidomain intervention program. Additional methodological details are available in the SUPERBRAIN study protocol [12].

### Outcomes

The primary outcome was the change in cognitive function, evaluated using the Repeatable Battery for the Assessment of Neuropsychological Status (RBANS) total scale index score from baseline to study completion [16]. The RBANS comprises 12 subtests used to assess five cognitive domains: immediate memory, visuospatial/constructional skills, language, attention, and delayed memory. Domain-specific index scores and the total scale index score (range: 40-160) are standardized to a mean of 100 with a standard deviation of 15, adjusted by age group for comparability [12, 17]. Early cognitive changes were defined as measurable declines in performance across these domains over a short period, as assessed using the RBANS [18].

Secondary outcomes included cognitive assessments such as the Mini-Mental State Examination (MMSE) and Clinical Dementia Rating (CDR), alongside evaluations of mood, daily functioning, quality of life, memory, nutrition, sleep quality, physical performance, and vascular risk factors. The assessment tools employed were the Bayer Activities of Daily Living Scale, Short Geriatric Depression Scale-Korean version (SGDS-K), Korean Quality of Life in AD (KQOL), Prospective Retrospective Memory Questionnaire (PRMQ), Nutrition Quotient for the Elderly (NQ-E), Mini Nutritional Assessment, Pittsburgh Sleep Quality Index, and Short Physical Performance Battery (SPPB). Trained assessors collected data at baseline and follow-up, while blinded outcome assessors ensured unbiased evaluations [12].

### Biomarker analysis

Blood samples were collected at baseline and study completion to measure critical biomarkers and evaluate metabolic and genetic factors. Brain-derived neurotrophic factor (BDNF) serum levels and GFAP plasma concentrations, neurofilament light chain (NfL), and pTau-181 were quantified employing Single Molecule Array (SIMOA) assays. BDNF levels were analyzed with the Simoa BDNF Discovery kit (Quanterix, Billerica, MA, PN/102039), GFAP and NfL levels were measured with the Simoa Neurology 2-Plex B kit (Quanterix, PN/103520), and pTau-181 levels were assessed utilizing the Simoa pTau-181 Advantage v2 kit (Quanterix, PN/103714). The assays were determined using the Simoa HD-X Analyzer® (Quanterix) through a two-step digital immunoassay procedure, following the protocol of the manufacturer. Genetic analysis involved extracting genomic DNA from blood samples and amplifying target regions using polymerase chain reaction for apolipoprotein E (APOE) genotyping. The presence of APOE alleles (ε2, ε3, and ε4) was identified to evaluate genetic susceptibility to AD.

### KBASE database and definition of β-amyloid positivity

Building on our recent publications from the KBASE-V cohort, the present study leveraged this cohort to further investigate the relationship between plasma p-tau181 levels and Centiloid values [13-15]. The KBASE-V cohort comprises a nationwide sample of cognitively unimpaired individuals, participants with MCI, and patients with AD recruited from nine hospitals across Republic of Korea between April 2015 and August 2016. All participants underwent comprehensive clinical evaluations, brain MRI, amyloid PET imaging (using 18F-flutemetamol or 11C-PiB), and blood biomarker analyses.

Amyloid deposition status was determined using a Centiloid threshold of ≥ 37, as previously established [19]. Plasma pTau-181 thresholds predictive of amyloid deposition were identified and subsequently applied in the present study to classify participants according to their predicted amyloid status.

### Ethics

This study complied with the principles of the Declaration of Helsinki and Good Clinical Practice guidelines. Ethical approval was obtained from the Institutional Review Boards (IRBs) of all participating centers, including Inha University Hospital (INHAUH-2021-06-040), Ewha Womans University Seoul Hospital (SEUMC-2021-07-037), Ewha Womans University Mokdong Hospital (EUMC-2021-08-003), Ajou University Hospital (AJIRB-BMR-SUR-21-323), Bobath Memorial Hospital (P01-202109-11-002), Dong-A University Hospital (DAUHIRB-21-168), Chonnam National University Hospital (CNUH-2021-326), Hanyang University Hospital (HYUH-2021-07-041), Myongji Hospital (MJH-2021-08-032), Jeonbuk National University Hospital (CUH-2021-08-043), Pusan National University Hospital (2108-016-015), Konkuk University Hospital (KUMC-2021-08-023), Samsung Medical Center (SMC-2021-08-022), CHA Bundang Medical Center (CHAMC-2022-01-020), Catholic Kwandong University International St. Mary’s Hospital (IS22EIMI0008), Daejeon Eulji Medical Center (EMC-2021-12-004), and Uijeongbu St. Mary’s Hospital (UIRB-2022-0428). All participants provided written informed consent following a comprehensive explanation of the study protocol. Personal data were anonymized, and unique study IDs were assigned to maintain confidentiality. Any protocol amendments were reviewed and approved by the relevant IRBs as required. An independent monitoring committee oversaw adverse events and study progress. This trial was registered on ClinicalTrials.gov (NCT05023057), and all procedures adhered to ethical standards for clinical research [12].

### Statistical methods

A post hoc exploratory subanalysis was conducted within the ITT population, which comprised all participants randomly assigned to the multidomain intervention or control group, irrespective of baseline assessment completion, intervention adherence, or follow-up participation. The analysis aimed to generate additional insights beyond the predefined analyses while preserving randomization integrity, ensuring group comparability, and minimizing bias attributed to differential loss to follow-up. Including all randomized participants enhanced the comprehensiveness of the intervention effect assessment, thereby improving the robustness and generalizability of the findings. Missing data were not imputed as the overall missing rate was relatively low (~10%) [11]. For biomarker-based subgroup analyses requiring plasma pTau-181 levels, a mITT approach was applied, including only participants (n = 276) with available baseline pTau-181 data (Fig. 1). This selective inclusion was necessitated by missing biomarker data, primarily due to unsuccessful blood sample collection, which was assumed to be missing at random. The mITT approach preserved group comparability while enabling a targeted evaluation of amyloid-related outcomes.

Correlation analyses were performed using the rcorr() function in R (Hmisc package), which applies pairwise deletion for handling missing data. This method computes correlations using all available data for each variable pair, thereby maximizing data retention and ensuring robust correlation estimates [20]. Correlations between continuous variables were visualized using scatter plots with linear regression lines. The relationships between plasma GFAP levels (baseline and 6-month follow-up) and the percentage change in RBANS scores over 6 months were evaluated using multivariable linear regression models. These analyses were adjusted for possible confounders, including sex, age, multidomain intervention status, APOE4 positivity, GFAP level (continuous variable in Table 2; categorical variable dichotomized at the median in Table 3), family history of dementia, hypertension, diabetes, hyperlipidemia, hypothyroidism, and medication use for AD. Additional stratified multivariable linear regression analyses were conducted among individuals with probable amyloid deposits to assess the associations between plasma GFAP levels and percentage change in RBANS total scale index scores, analyzed separately for the standard care and multidomain intervention groups. To assess the robustness of the regression models, model fit and multicollinearity were evaluated. Sensitivity analyses were conducted to validate the findings. Missing data were handled using multiple imputations, assuming the data were missing at random. The imputation model included age, sex, group assignment, the percentage change in RBANS scores, and plasma GFAP levels at baseline and 6 months as predictors. Missing values were imputed employing the fully conditional specification method with regression modeling, generating 10 imputed datasets. Rubin’s rules were applied to pool estimates across datasets. Rubin’s rules combine estimates from multiple imputed datasets by averaging parameters and adjusting standard errors, accounting for both within- and between-imputation variability, thus providing valid statistical inferences [21].

Amyloid-beta plaque positivity was predicted based on baseline plasma pTau181 levels, with the optimal cutoff derived from receiver operating characteristic (ROC) curve analysis using data from the KBASE-V cohort. Amyloid positivity was defined using a Centiloid threshold of ≥ 37 [18, 19].

Statistical significance was set at p < 0.05. Graphical outputs were generated using the ggplot2 package in R (version 4.3.3) and IBM SPSS Statistics for Windows (version 24.0; IBM Corp., Armonk, NY, USA).

## RESULTS

### Participant characteristics

Between August 2021 and June 2022, 309 individuals were screened for eligibility, and 300 elderly participants (aged 60-85 years) with MCI were randomly categorized in this prospective, multicenter RCT (Fig. 1). Overall, 148 participants (49.3%) underwent the multidomain intervention. The mean participant age was 73.0 years, with 70.7% being female. The most prevalent APOE genotype was E3/E3 (57.7%), followed by E3/E4 (27.0%) and E2/E3 (8.3%) (Table 1). Table 1 summarizes additional information regarding participant characteristics.

### Changes in RBANS, secondary outcome measures, and blood biomarkers over a 6-month follow-up period

The RBANS score increased from 87.7 at baseline to 95.3 after 6 months (p < 0.001) (Supplementary Table 1). Significant improvements were observed across multiple RBANS subdomains, including immediate memory, visuospatial abilities, language, and delayed memory. Supplementary Fig. 1 illustrates changes in RBANS total score and its five cognitive domains at baseline, 12 weeks, and 24 weeks, stratified by standard care and multidomain intervention groups. Overall, cognitive scores consistently increased over time in both groups, with significantly greater improvements observed in the multidomain intervention group, suggesting enhanced cognitive performance. Additionally, statistically significant improvements were observed in other assessments, including the SGDS-K, KQOL, NQ-E, and SPPB, over the same period (Supplementary Table 1). Significant changes were also observed in blood biomarkers, including NfL and BDNF levels, whereas GFAP and pTau-181 levels remained unchanged (Supplementary Table 1). Individual-level changes in blood biomarker levels between baseline and after 6 months of follow-up were visualized using boxplots (Supplementary Fig. 2).

**Table 1:** Demographic and clinical characteristics of study participants with MCI.

Patients’ characteristics	Multidomain intervention (-)	Multidomain intervention (+)	Total	P
Number, n (%)	152 (50.7)	148 (49.3)	300	
Sex, female, n (%)	115 (75.7)	97 (65.5)	212 (70.7)	0.058
Age, mean ± SD, (years)	73.1 ± 5.6	72.9 ± 5.5	73.0 ± 5.6	0.812
APOE genotype, n (%)				0.579
E2/E2	4 (2.6)	2 (1.4)	6 (2.0)	
E2/E3	11 (7.2)	14 (9.5)	25 (8.3)	
E2/E4	3 (2.0)	1 (0.7)	4 (1.3)	
E3/E3	92 (60.5)	81 (54.7)	173 (57.7)	
E3/E4	38 (25.0)	43 (29.1)	81 (27.0)	
E4/E4	4 (2.6)	7 (4.7)	11 (3.7)	
APOE4 positivity	45 (29.6)	51 (34.5)	96 (32.0)	0.388
Medical History, n (%)				
Family history of dementia	42 (27.6)	52 (35.1)	94 (31.3)	0.173
Hypertension	72 (47.4)	67 (45.3)	139 (46.3)	0.730
Diabetes	34 (22.4)	40 (27.0)	74 (24.7)	0.422
Hyperlipidemia	95 (62.5)	99 (66.9)	194 (64.7)	0.469
Hypothyroidism	2 (1.3)	8 (5.4)	10 (3.3)	0.058
Medication for AD (cholinesterase inhibitor or NMDA receptor antagonists), n (%)	32 (21.1)	37 (25.0)	69 (23.0)	0.493

Abbreviations: AD, Alzheimer's disease; APOE, apolipoprotein E; MCI, mild cognitive impairment; NMDA, N-Methyl-D-Aspartate; SD, standard deviation.

### Associations between plasma GFAP levels, multidomain intervention, and changes in cognitive performance over 6 months

**Figure 2.**
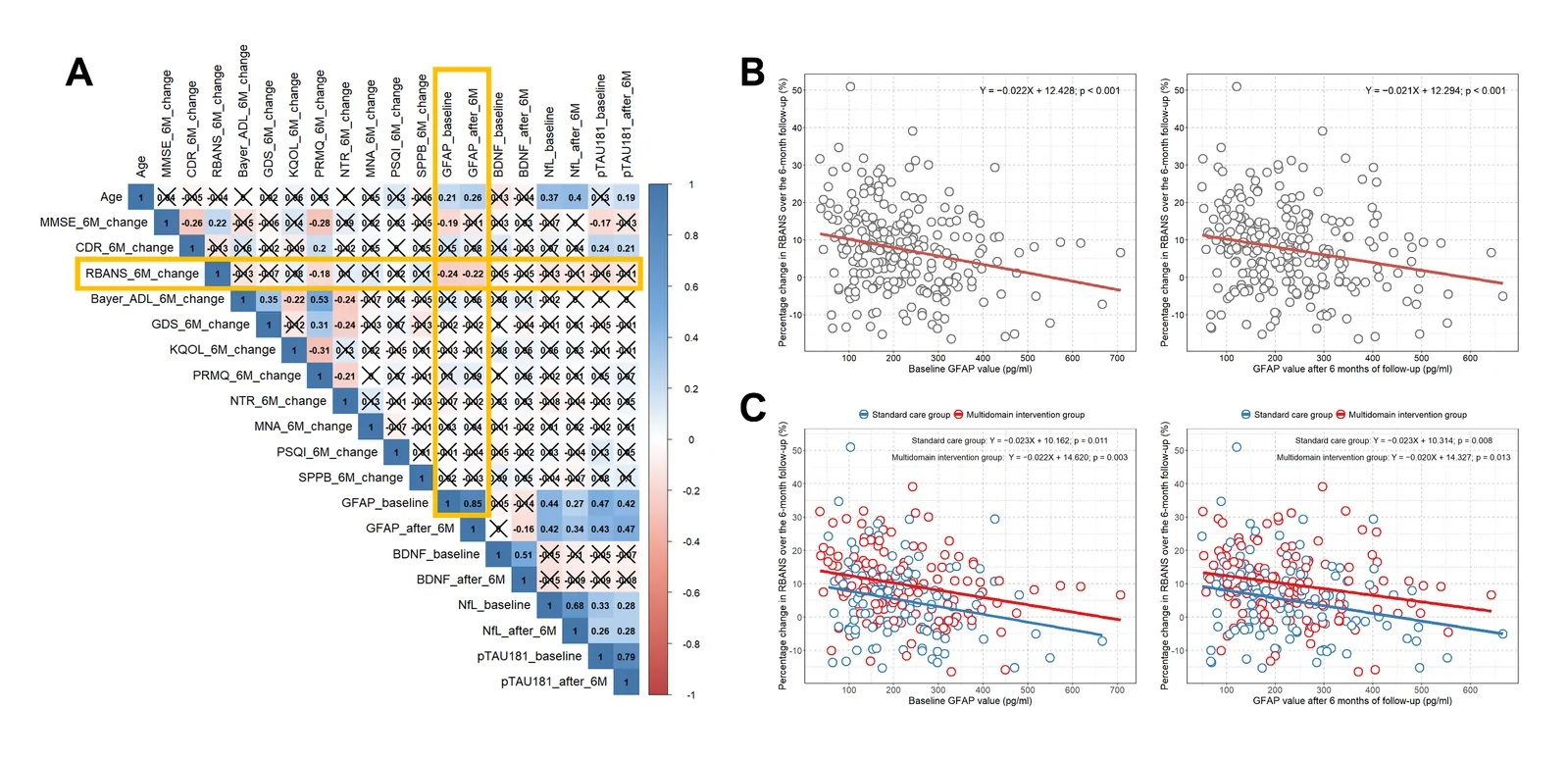
**Pearson correlation coefficients and associations between plasma GFAP levels and changes in RBANS scores over 6 months**. (A) Correlation matrix showing Pearson correlation coefficients among cognitive measures, secondary outcomes, and plasma biomarkers at baseline and after 6 months. The color-coded scale indicates the strength and direction of the correlation coefficients, with blue and red shades representing positive and negative correlations, respectively. An “x” indicates nonsignificant relationships (p ≥ 0.001. (B) Scatterplots with linear regression lines displaying the relationship between plasma GFAP levels (pg/mL) and percentage change in RBANS scores at 6 months. The left panel illustrates the relationship between baseline plasma GFAP levels and percentage changes in RBANS scores, while the right panel illustrates the correlation between plasma GFAP levels after 6 months and percentage changes in RBANS scores. (C) Scatterplots with linear regression lines showing the relationship between plasma GFAP levels and percentage changes in RBANS scores, stratified by group: standard care (blue) and multidomain intervention (red). The left panel illustrates the relationship between baseline plasma GFAP levels and percentage changes in RBANS scores, while the right panel illustrates the association between plasma GFAP levels after 6 months and percentage changes in RBANS scores. Abbreviations: ADL, activities of daily living; BDNF, brain-derived neurotrophic factor; CDR, Clinical Dementia Rating; GFAP, glial fibrillary acidic protein; KQOL, Korean Quality Of Life; MMSE, Mini-Mental State Examination; MNA, Mini Nutritional Assessment; NfL, neurofilament light chain; NQ-E, Nutrition Quotient for the Elderly; PRMQ, Prospective And Retrospective Memory Questionnaire; PSQI, Pittsburgh Sleep Quality Index; pTau-181, phosphorylated tau 181; RBANS, Repeatable Battery for the Assessment of Neuropsychological Status; SPPB, Short Physical Performance Battery.

We analyzed the correlations between the percentage changes in RBANS scores, secondary outcome measures, and blood biomarker levels at baseline and after 6 months. Among the biomarkers assessed, GFAP levels at both time points demonstrated statistically significant negative correlations with the percentage change in RBANS scores over the 6-month follow-up period (baseline: β = -0.24, p < 0.001; 6 months: β = -0.22, p < 0.001) (Fig. 2A). As GFAP levels increased at baseline and 6 months, the percentage change in RBANS score over 6 months significantly decreased across all participants (Fig. 2B). In the multidomain intervention and standard care groups, GFAP levels at baseline and 6 months remained significantly and negatively correlated with the percentage change in RBANS scores over 6 months, with similar regression slopes (Fig. 2C). Higher baseline and 6-month GFAP levels were independently and negatively associated with the percentage changes in RBANS scores over 6 months in elderly participants with MCI (baseline: β = -0.019; p = 0.004; 6 months: β = -0.018; p = 0.007) (Table 2). In addition, participation in the multidomain intervention was independently associated with significantly greater improvements in the percentage change in RBANS scores over 6 months, with β values ranging from 5.053-5.057 depending on the model specification (Table 2). Sensitivity analyses (Supplementary Table 2) confirmed the robustness of the primary findings (Table 2).

### Determination of the optimal plasma pTau-181 threshold for predicting amyloid β plaque deposition

Supplementary Table 3 summarizes the characteristics of the participants from the KBASE-V cohort. The mean age was 69.8 years, and 58.6% were female. The mean baseline Centiloid value was 18.6, and 24.7% of participants were classified as amyloid positive (Centiloid ≥ 37). The mean baseline plasma pTau-181 concentration was 2.96 pg/mL. Using data from the KBASE-V cohort, we examined the relationship between plasma pTau-181 levels and amyloid burden, quantified via Centiloid values (Supplementary Fig. 3A). Linear regression analysis revealed a significant positive association between plasma pTau-181 concentrations and Centiloid values. ROC curve analysis revealed an optimal plasma pTau-181 cutoff value of 2.940 pg/mL for predicting amyloid β plaque positivity, defined by a Centiloid threshold of ≥ 37 (AUC = 0.889; sensitivity = 90.7%; specificity = 82.4%) (Supplementary Fig. 3B). This threshold was subsequently applied to classify participants based on their predicted amyloid status, facilitating further analyses on the effects of multidomain intervention and plasma GFAP levels on cognitive outcomes over 6 months.

**Table 2:** Multivariable linear regression analysis of percentage changes in RBANS scores over 6 months, separately including baseline GFAP levels or 6-month GFAP levels as independent variables.

Multivariable linear regression analysis				
**All study participants (n = 300)**				
	**Baseline GFAP levels**		**6-month GFAP levels**	
Variable	β (95% CI)	*p*-value	β (95% CI)	*p*-value
Sex (female)	1.017 (-2.00 to 4.03)	0.507	1.138 (-1.92 to 4.20)	0.465
Age (years)	-0.001 (-0.27 to 0.27)	0.993	-0.002 (-0.28 to 0.27)	0.986
Multidomain intervention	**5.057(2.37 to 7.75)**	< **0.001**	**5.053(2.33 to 7.78)**	< **0.001**
APOE4 positivity	-1.050 (-4.06 to 1.96)	0.493	-1.119 (-4.17 to 1.93)	0.470
GFAP levels	**-0.019 (-0.031 to -0.006)**	**0.004**	**-0.018 (-0.030 to -0.005)**	**0.007**
Family history of dementia	-1.203 (-4.12 to 1.71)	0.417	-1.231 (-4.18 to 1.72)	0.412
Hypertension	0.960 (-1.81 to 3.73)	0.495	1.034 (-1.77 to 3.84)	0.469
Diabetes	0.138 (-3.06 to 3.33)	0.932	0.236 (-3.02 to 3.49)	0.887
Hyperlipidemia	-1.168 (-3.96 to 1.62)	0.410	-1.107 (-3.96 to 1.74)	0.445
Hypothyroidism	-0.118 (-7.39 to 7.16)	0.975	-0.420 (-7.77 to 6.93)	0.910
Medication for AD	-2.719 (-5.98 to 0.54)	0.102	-2.997 (-6.26 to 0.27)	0.072

Abbreviations: AD, Alzheimer's disease; APOE4, apolipoprotein E4; CI, confidence interval; GFAP, glial fibrillary acidic protein; RBANS, Repeatable Battery for the Assessment of Neuropsychological Status.

**Table 3:** Multivariable linear regression analysis of percentage changes in RBANS total scale index scores over 6 months, based on the modified ITT population with available plasma pTau181 data.

Multivariable linear regression analysis				
**Participants classified as probable amyloid-negative or amyloid-positive (n = 276)**				
	Probable amyloid-negative group (n = 173)		Probable amyloid-positive group (n = 103)	
Variable	β (95% CI)	*p*-value	β (95% CI)	*p*-value
Sex (female)	0.227 (-3.77 to 4.23)	0.911	2.565 (-2.43 to 7.56)	0.310
Age (years)	-0.233 (-0.59 to 0.12)	0.197	0.186 (-0.25 to 0.63)	0.403
Multidomain intervention	**3.976 (0.42 to 7.54)**	**0.029**	**5.983 (1.19 to 10.78)**	**0.015**
APOE4 positivity	-0.255 (-4.65 to 4.14)	0.909	-1.514 (-6.28 to 3.25)	0.529
High baseline GFAP group (above median)	-1.437 (-5.46 to 2.58)	0.481	-4.522 (-9.98 to 0.93)	0.103
Family history of dementia	-0.989 (-4.77 to 2.79)	0.606	-1.169 (-6.30 to 3.96)	0.651
Hypertension	0.804 (-2.86 to 4.47)	0.666	0.772 (-3.94 to 5.49)	0.745
**Diabetes**	-0.621 (-4.81 to 3.57)	0.770	1.219 (-4.71 to 7.15)	0.684
Hyperlipidemia	-0.590 (-4.36 to 3.18)	0.757	-0.485 (-5.25 to 4.28)	0.840
Hypothyroidism	-3.297 (-14.83 to 8.23)	0.573	3.307 (-6.50 to 13.11)	0.504
Medication for AD	-1.489 (-6.43 to 3.45)	0.552	-3.629 (-8.55 to 1.30)	0.146
**Individuals with probable amyloid deposit (n = 103)**				
	Standard care group (n = 51)		Multidomain intervention group (n = 52)	
Variable	β (95% CI)	*p*-value	β (95% CI)	*p*-value
Sex (female)	3.296 (-5.60 to 12.19)	0.456	2.258 (-4.40 to 8.91)	0.496
Age (years)	-0.098 (-0.76 to 0.56)	0.765	0.500 (-0.19 to 1.19)	0.151
APOE4 positivity	-1.134 (-8.92 to 6.66)	0.769	0.849 (-6.79 to 8.49)	0.823
High baseline GFAP group (above median)	-0.437 (-9.36 to 8.49)	0.921	**-8.661 (-16.91 to -0.41)**	**0.040**
Family history of dementia	-2.848 (-11.33 to 5.63)	0.499	0.817 (-6.36 to 7.99)	0.819
Hypertension	3.907 (-4.10 to 11.92)	0.329	-0.538 (-8.66 to 7.58)	0.894
Diabetes	1.738 (-10.08 to 13.55)	0.767	0.887 (-7.34 to 9.12)	0.828
Hyperlipidemia	0.083 (-7.87 to 8.03)	0.983	-0.776 (-7.65 to 6.10)	0.820
Hypothyroidism	15.001 (-8.72 to 38.72)	0.207	-1.687 (-13.27 to 9.89)	0.769
Medication for AD	-6.986 (-14.23 to 0.26)	0.058	0.335 (-7.27 to 7.94)	0.929

Analyses were performed for participants classified as probable amyloid-negative or probable amyloid-positive and for individuals with probable amyloid positivity stratified by standard care and multidomain intervention groups. Abbreviations: AD, Alzheimer's disease; APOE4, apolipoprotein E4; CI, confidence interval; GFAP, glial fibrillary acidic protein; ITT, intention-to-treat; RBANS, Repeatable Battery for the Assessment of Neuropsychological Status.

**Figure 3.**
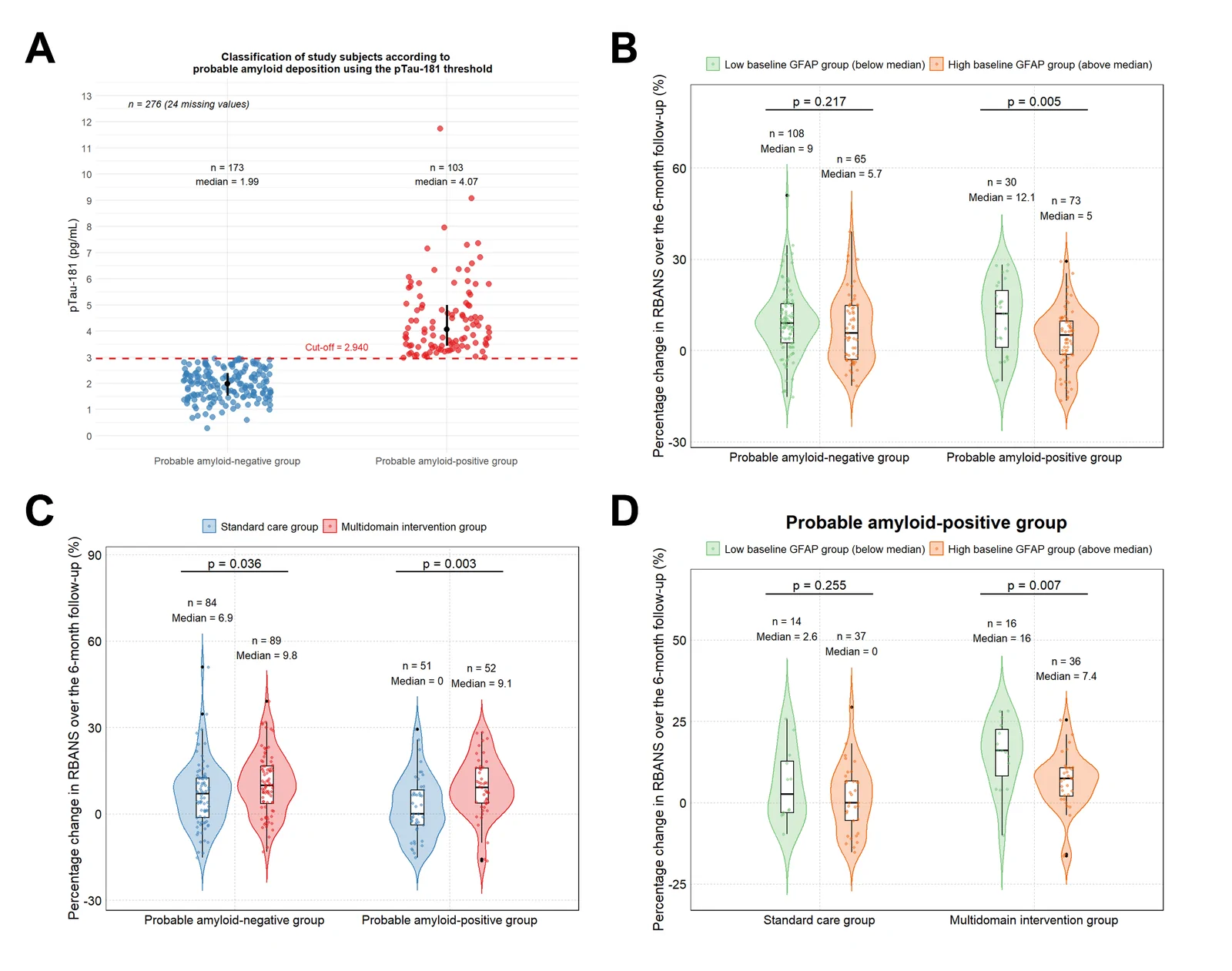
**Cognitive effects of multidomain intervention and predictive value of baseline plasma GFAP levels, stratified by probable amyloid-β deposition**. (**A**) Classification of participants into probable amyloid-negative and probable amyloid-positive groups based on a plasma pTau-181 cutoff of 2.940 pg/mL. Each dot represents an individual plasma pTau-181 level. (**B**) Violin plots illustrating the percentage change in RBANS scores over 6 months, stratified by baseline plasma GFAP levels (below vs. above median) within probable amyloid-negative and probable amyloid-positive groups. (**C**) Violin plots showing the percentage change in RBANS scores comparing standard care and multidomain intervention groups within probable amyloid-negative and probable amyloid-positive groups. (**D**) Violin plots illustrating the effect of baseline plasma GFAP levels (below vs. above median) within the standard care and multidomain intervention groups among probable amyloid-positive groups. Abbreviations: GFAP, glial fibrillary acidic protein; pTau-181, phosphorylated tau 181; RBANS, Repeatable Battery for the Assessment of Neuropsychological Status.

### Cognitive benefits of multidomain intervention and predictive value of baseline plasma GFAP in individuals stratified by probable amyloid-β plaque deposition

In our cohort, 276 participants with available plasma pTau-181 measurements were classified into probable amyloid-negative (n =173) and probable amyloid-positive (n = 103) groups based on a pTau-181 cutoff of 2.940 pg/mL (Fig. 3A). Baseline plasma GFAP levels were significantly higher in the probable amyloid-positive group than those in the probable amyloid-negative group (Supplementary Fig. 4). However, no significant difference was observed between both groups in the percentage change in GFAP levels over the 6-month follow-up period. Among the probable amyloid-positive group, participants with lower baseline GFAP levels (below median) showed significantly greater improvements in RBANS scores over 6 months than those with higher baseline GFAP levels (above median) (Fig. 3B).

In contrast, no significant difference in RBANS score changes was observed in the probable amyloid-negative group according to baseline GFAP levels. The multidomain intervention group showed significantly greater improvements in RBANS scores over 6 months than that of the standard care group in participants with probable amyloid-negative (p = 0.036) and probable amyloid-positive (p = 0.003) (Fig. 3C). Moreover, the multidomain intervention exhibited statistically significant improvements in several secondary outcome domains, such as global cognitive function (MMSE), dementia severity (CDR), depressive symptoms (SGDS-K), subjective memory complaints (PRMQ), and nutritional status (NQ-E), although the pattern of significance varied by amyloid status (Supplementary Fig. 5). These findings suggest broad beneficial effects of the intervention beyond the improvements observed in RBANS scores. When analyses were restricted to individuals with probable amyloid positivity, baseline GFAP levels predicted cognitive response to the intervention. Among participants who underwent multidomain intervention, those with low baseline GFAP levels exhibited significantly greater cognitive improvements than those with high baseline GFAP levels (p = 0.007) (Fig. 3D). However, no such relationship was observed in the standard care group.

Multivariable linear regression analyses further supported these findings (Table 3). In probable amyloid-negative and probable amyloid-positive groups, multidomain intervention was independently associated with greater RBANS score improvements over 6 months after adjusting for possible confounders. Among participants with probable amyloid positivity who underwent the multidomain intervention, classification in the high baseline GFAP group was significantly associated with reduced cognitive improvement compared to that in the low baseline GFAP group (β = -8.661, p = 0.040) (Table 3).

Sensitivity analyses, conducted by applying the same multivariable linear regression models to datasets generated through Rubin’s multiple imputation method, yielded consistent results (Supplementary Tables 4 and 5). In these sensitivity analyses, the multidomain intervention remained significantly associated with greater improvements in RBANS scores in amyloid-negative and amyloid-positive groups. Moreover, among individuals with probable amyloid positivity who underwent multidomain intervention, higher baseline GFAP levels continued to be significantly associated with diminished cognitive improvement, further reinforcing the robustness of our primary findings.

## DISCUSSION

This study demonstrates that among various plasma biomarkers, plasma GFAP levels—at baseline and after 6 months—are significantly and negatively associated with short-term cognitive improvement, as reflected by the percentage change in RBANS scores, in elderly individuals with MCI, regardless of whether they underwent the multidomain intervention or standard care. These findings suggest that elevated plasma GFAP levels may serve as a predictive biomarker for reduced cognitive gains over a short period, even in the context of multidomain interventions or standard symptomatic care. When analyzing the study population according to probable amyloid deposition status, the negative effect of elevated baseline plasma GFAP levels on cognitive improvement was statistically significant only in individuals with probable amyloid deposition, suggesting a specific interaction between underlying amyloid pathology and astroglial activation. In addition, the multidomain intervention over 6 months resulted in significant improvements in global cognitive function, as measured via RBANS scores, in the probable amyloid-negative and probable amyloid-positive groups. These findings prompted us to further investigate whether baseline plasma GFAP levels could differentially predict cognitive responses to multidomain interventions among individuals with probable amyloid pathology. In the probable amyloid-positive group, participants with higher baseline GFAP levels exhibited significantly reduced cognitive improvements following the multidomain intervention compared to those with lower baseline GFAP levels. Collectively, these findings suggest that in individuals with underlying amyloid pathology, baseline plasma GFAP levels may serve as a predictive biomarker for short-term cognitive response to multidomain interventions aimed at preserving cognitive function. To our knowledge, this is the first clinical evidence from an RCT suggesting that elevated baseline plasma GFAP levels in individuals with amyloid deposition are associated with diminished short-term cognitive benefits from multidomain interventions. These findings highlight the potential importance of considering astroglial activation alongside amyloid pathology when predicting therapeutic responsiveness in elderly individuals with MCI.

The RBANS served as the primary tool for assessing cognitive outcomes in this study. It is particularly effective for detecting subtle and rapid cognitive decline in individuals with MCI or early AD [22, 23]. The RBANS provides a comprehensive evaluation across five key cognitive domains—immediate memory, visuospatial/constructional abilities, language, attention, and delayed memory—enabling a detailed profile of domain-specific impairments. This level of granularity distinguishes it from broader tools, including the MMSE [23]. Additionally, the RBANS demonstrates high sensitivity in identifying early cognitive changes, differentiating MCI from normal aging, and tracking longitudinal progression, making it ideal for assessing intervention outcomes [23, 24]. Furthermore, it is culturally adaptable and supported by robust normative data across age groups and populations, which improves its accuracy and minimizes potential biases observed in other assessments [25]. These features make the RBANS a reliable and versatile tool for early cognitive assessment in clinical practice and research.

GFAP, a well-established marker of reactive astrogliosis, is a crucial cytoskeletal protein in astrocytes and is associated with neuronal injury and neurodegeneration [26]. Elevated plasma GFAP levels indicate astrocytic activation in response to early neuronal injury and they are linked to subtle cognitive decline, even during the prodromal stages of cognitive impairment [26, 5, 27]. Given that our study population consisted of individuals with MCI, assessing plasma GFAP levels combined with sensitive cognitive measures such as the RBANS is appropriate for identifying those at risk of early AD progression. This strategy is supported by evidence showing that RBANS and plasma GFAP levels are effective in detecting early cognitive decline during the prodromal stages of cognitive impairment.

This study demonstrates that baseline astroglial activation, reflected by plasma GFAP concentrations, plays a critical role in modulating cognitive responsiveness, even over relatively short follow-up periods. This relationship was particularly evident among participants with underlying amyloid deposition, suggesting that astrocyte reactivity may interact with amyloid pathology to reduce cognitive resilience. Building on these findings, the attenuated cognitive improvements observed in individuals with probable amyloid positivity with elevated baseline plasma GFAP levels, despite undergoing multidomain interventions, may be due to a synergistic pathological interaction between amyloid deposition and astroglial activation. In the presence of amyloid pathology, astrocyte activation—as indicated by elevated plasma GFAP levels—may amplify neuroinflammatory responses and promote the development of a neurotoxic microenvironment, thereby impairing neuronal plasticity and limiting the capacity of the brain for cognitive recovery [6, 7, 28, 29]. This neuroinflammatory amplification may diminish the potential benefits of multidomain interventions aimed at preserving cognitive function. Furthermore, among individuals with probable amyloid deposition, those with lower baseline plasma GFAP levels showed more favorable short-term cognitive responses to these interventions. These findings suggest that, although amyloid pathology is present, relatively astroglial activation may reflect a less advanced neuroinflammatory state, thereby preserving neuronal resilience and synaptic plasticity essential for short-term cognitive recovery. Previous studies report that astrocyte reactivity and associated neuroinflammation exacerbate neurodegeneration in the context of amyloid pathology, whereas lower levels of astrocytic activation may mitigate the neurotoxic effects of amyloid deposition [28, 30]. Accordingly, individuals with amyloid pathology but lower baseline GFAP levels may retain greater neurobiological reserve, enabling more effective short-term cognitive recovery following multidomain interventions [31].

Recent studies have highlighted a robust association between plasma GFAP levels and amyloid-β accumulation, supporting the utility of plasma GFAP not only as a marker of reactive astrocytosis but also as an indirect biomarker of cerebral amyloid pathology [8, 9]. For instance, Benedet et al. demonstrated that plasma GFAP explained a significant proportion of amyloid-PET signal variance across the Alzheimer's disease continuum, even outperforming CSF GFAP in distinguishing amyloid-positive individuals [8]. This accumulating evidence suggests that plasma GFAP may reflect an astroglial response that is closely linked to amyloid burden, albeit with notable inter-individual variability [9]. The presence of this low-GFAP/amyloid-positive subgroup implies pathophysiological heterogeneity. In our findings, the enhanced cognitive benefit of multidomain interventions among amyloid-positive individuals with lower baseline plasma GFAP levels may indicate a biologically distinct subgroup characterized by early-stage or less reactive astroglial activation. This subgroup might retain greater neuroplasticity or exhibit reduced neuroinflammation, thereby responding more favorably to lifestyle-based interventions [32]. Clarifying the biological features that define this phenotype warrants further investigation and could inform biomarker-guided, personalized strategies for mitigating cognitive decline in MCI and early AD [33].

Although this study provides valuable insights, several limitations should be acknowledged. First, the absence of long-term follow-up data on cognitive outcomes and blood biomarkers limits our understanding of the trajectory of cognitive decline and the potential durability of intervention effects. Second, amyloid pathology was inferred from plasma pTau-181 levels rather than directly confirmed through amyloid PET imaging, which may introduce misclassification bias. Third, the cohort consisted exclusively of elderly Korean individuals with MCI, which may limit the generalizability of our findings to other ethnicities and broader clinical populations. Fourth, although not statistically significant, a modest imbalance in sex distribution between study arms (female proportion 75.7% vs 65.5%) could potentially influence outcomes. Sex was therefore included as a possible confounder in all multivariable models to mitigate any residual bias. Finally, the study assessed only plasma biomarkers without incorporating cerebrospinal fluid analyses, which could provide additional insights into central nervous system pathology.

## Conclusions

This multicenter RCT highlights plasma GFAP as a potential early biomarker for cognitive stage transitions in elderly individuals with MCI. Our findings suggest that, among individuals with probable amyloid pathology, baseline plasma GFAP levels may reflect disease severity and predict cognitive response to therapeutic interventions. These findings underscore the importance of assessing amyloid burden and astrocytic activation when forecasting therapeutic outcomes in elderly individuals with MCI. Future studies incorporating amyloid PET imaging and longer-term follow-up should validate these findings, supporting personalized therapeutic strategies for at-risk populations.

## Supplementary Materials

The Supplementary data can be found online at: www.aginganddisease.org/EN/10.14336/AD.2025.0646.



## Data Availability

The data supporting the findings of this study are available on request from the corresponding author.
