# Supplementary material for: Plasma GFAP and Amyloid Pathology Predict Cognitive Response to Multidomain Interventions in MCI
Source: Aging Dis. 2025 Jun 29;17(4):2260–72. doi: 10.14336/AD.2025.0646 (PMC13256596; doi:10.14336/AD.2025.0646)
Supplement: Supplementary file 1 [file AD-17-4-2260-s.pdf]

SUPPLEMENTARY DATA

Plasma GFAP and Amyloid Pathology Predict Cognitive Response to Multidomain Interventions in MCI

Myung-Hoon Han, Mina Hwang, Hyuk Sung Kwon, So Young Moon, Yoo Kyoung Park, Jee Hyang Jeong, Seong Hye Choi, Seong-Ho Koh

© 2025. Han MH et al. Published online at http://www.aginganddisease.org/EN/10.14336/AD.2025.0645

SUPPLEMENTARY DATA

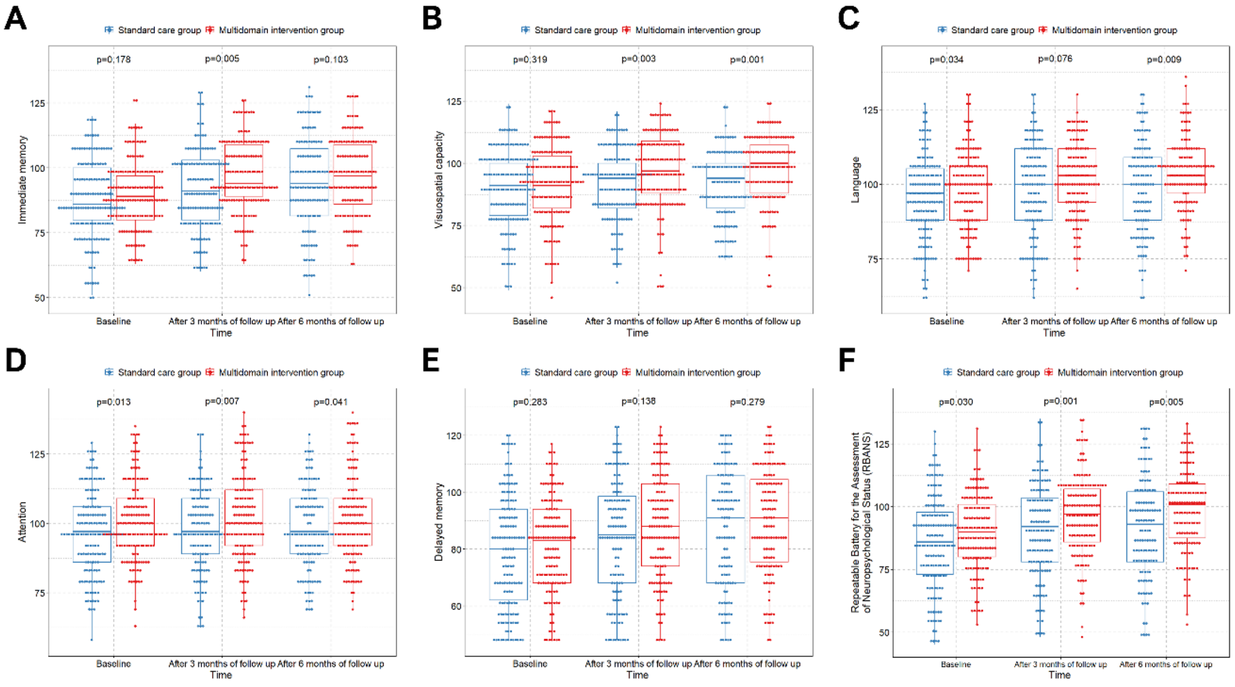

Supplementary Figure 1. Boxplots illustrating changes in RBANS subdomain and total scores over time, stratified by group: standard care (blue) and multidomain intervention (red). (A) Immediate memory scores at baseline, 3 months, and 6 months. (B) Visuospatial capacity scores at baseline, 3 months, and 6 months. (C) Language scores at baseline, 3 months, and 6 months. (D) Attention scores at baseline, 3 months, and 6 months. (E) Delayed memory scores at baseline, 3 months, and 6 months. (F) Total RBANS scores at baseline, 3 months, and 6 months.

Abbreviation: RBANS, Repeatable Battery for the Assessment of Neuropsychological Status.

© 2025. Han MH et al. Published online at http://www.aginganddisease.org/EN/10.14336/AD.2025.0645

SUPPLEMENTARY DATA

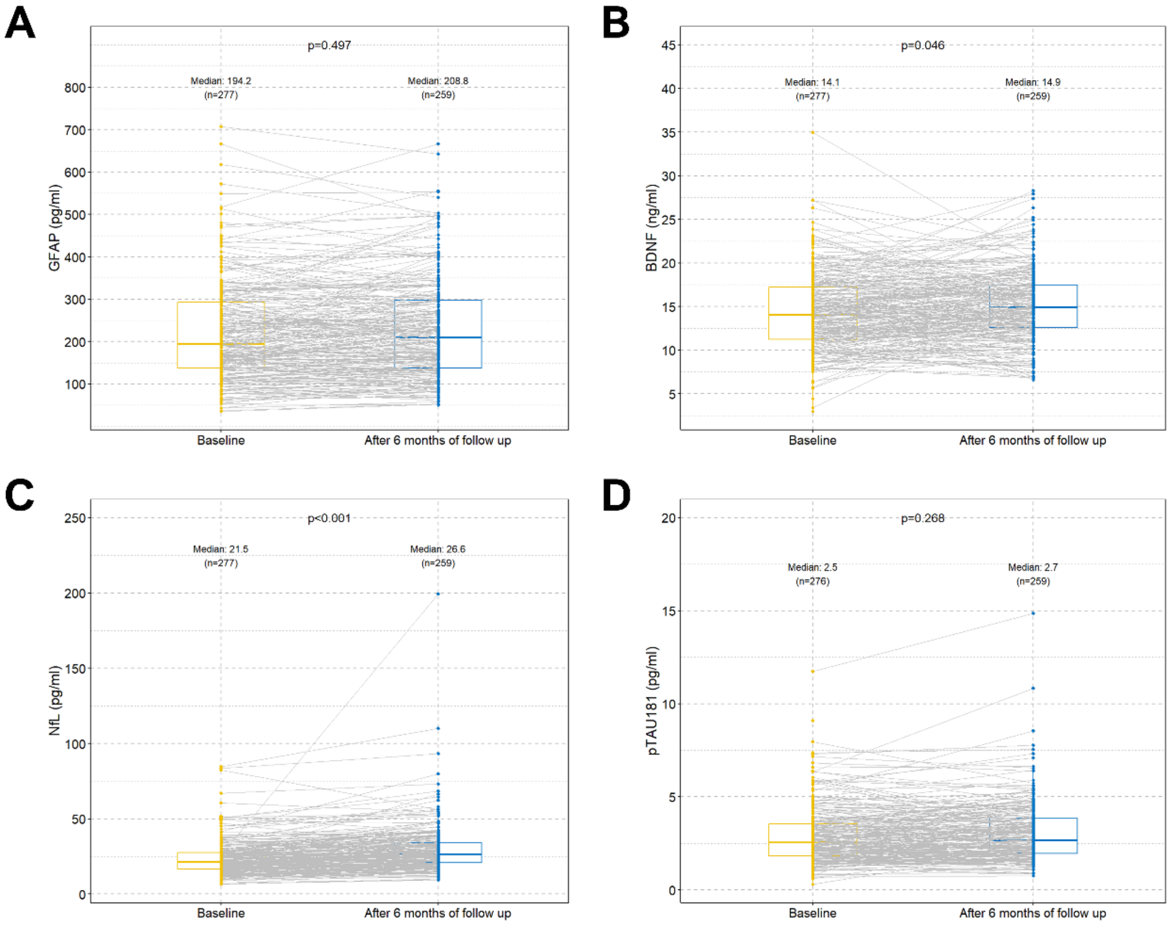

Supplementary Figure 2. Paired comparisons of plasma biomarker levels at baseline (yellow) and 6 months (blue) for each participant. (A) Plasma GFAP levels (pg/mL), with no significant change between baseline and 6 months. (B) Plasma BDNF levels (ng/mL) showing a significant decrease over 6 months. (C) Plasma NfL levels (pg/mL) showing a significant increase over 6 months. (D) Plasma pTau181 levels (pg/mL), with no significant change over 6 months.

Abbreviations: BDNF, brain-derived neurotrophic factor; GFAP, glial fibrillary acidic protein; NfL, neurofilament light chain; pTau181, phosphorylated tau 181.

SUPPLEMENTARY DATA

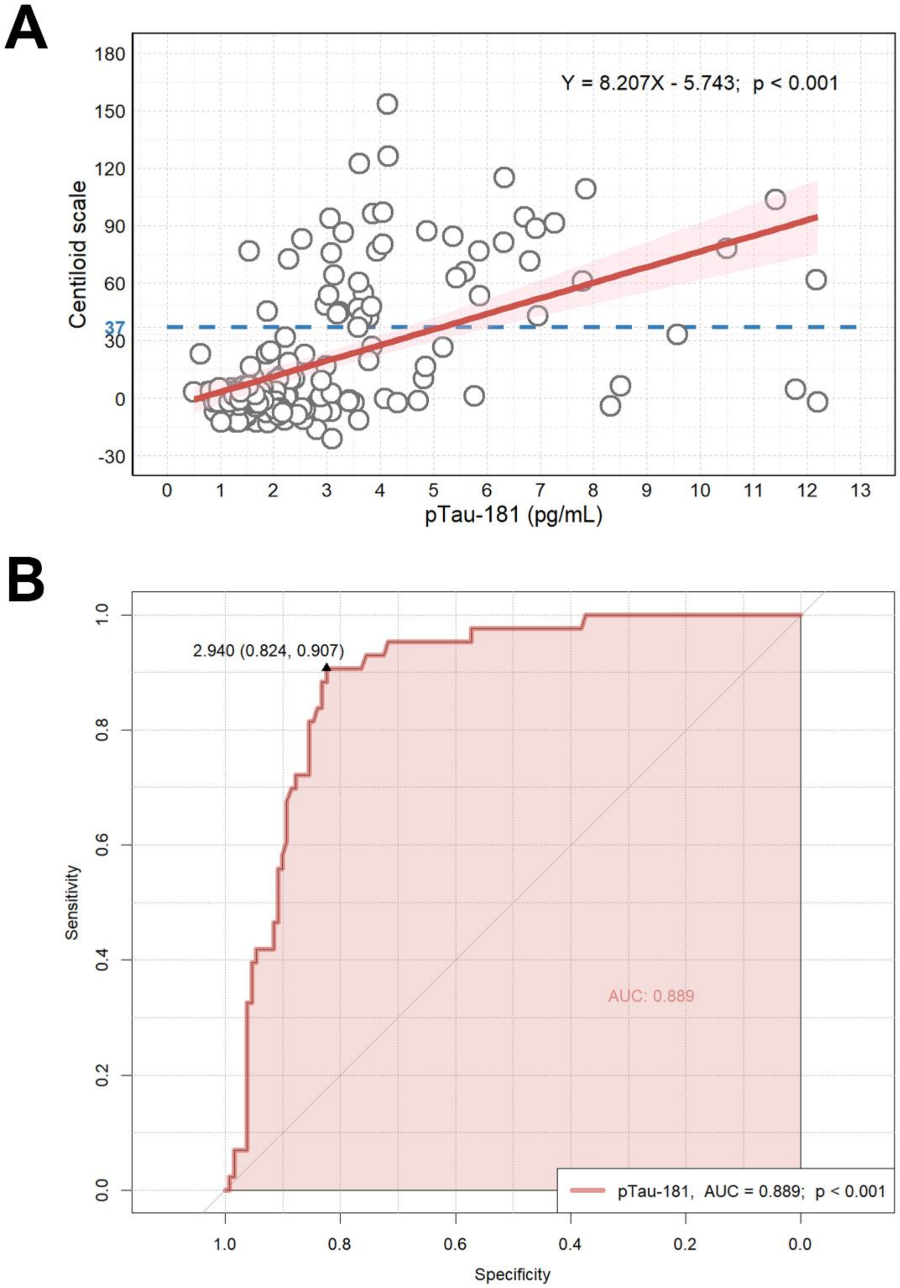

Supplementary Figure 3. Plasma pTau-181 levels in relation to amyloid burden and diagnostic performance for predicting amyloid positivity. (A) Scatter plot illustrating a positive linear correlation between plasma pTau-181 levels (pg/mL) and amyloid burden, quantified using Centiloid scale values. The red line represents regression, and the blue dashed line indicates the Centiloid threshold of 37 used to define β-amyloid. (B) ROC curve for plasma pTau-181 levels predicting β-amyloid positivity (Centiloid ≥ 37).

Abbreviations: AUC, area under the curve; pTau-181, phosphorylated tau 181; ROC, receiver operating characteristic.

© 2025. Han MH et al. Published online at http://www.aginganddisease.org/EN/10.14336/AD.2025.0645

SUPPLEMENTARY DATA

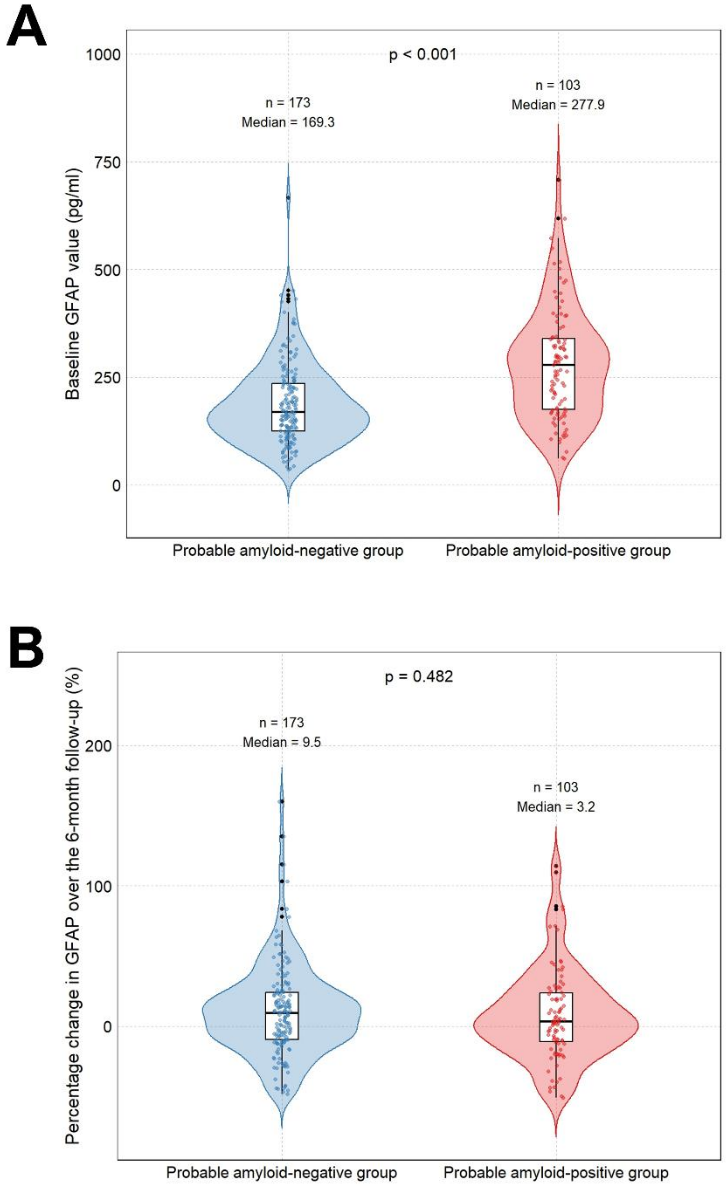

Supplementary Figure 4. Boxplots within violin plots illustrating plasma GFAP measurements stratified by probable amyloid-negative and probable amyloid-positive groups. (A) Baseline GFAP levels (pg/mL). (B) Percentage change in GFAP levels over 6 months.

Abbreviation: GFAP, glial fibrillary acidic protein.

© 2025. Han MH et al. Published online at http://www.aginganddisease.org/EN/10.14336/AD.2025.0645

SUPPLEMENTARY DATA

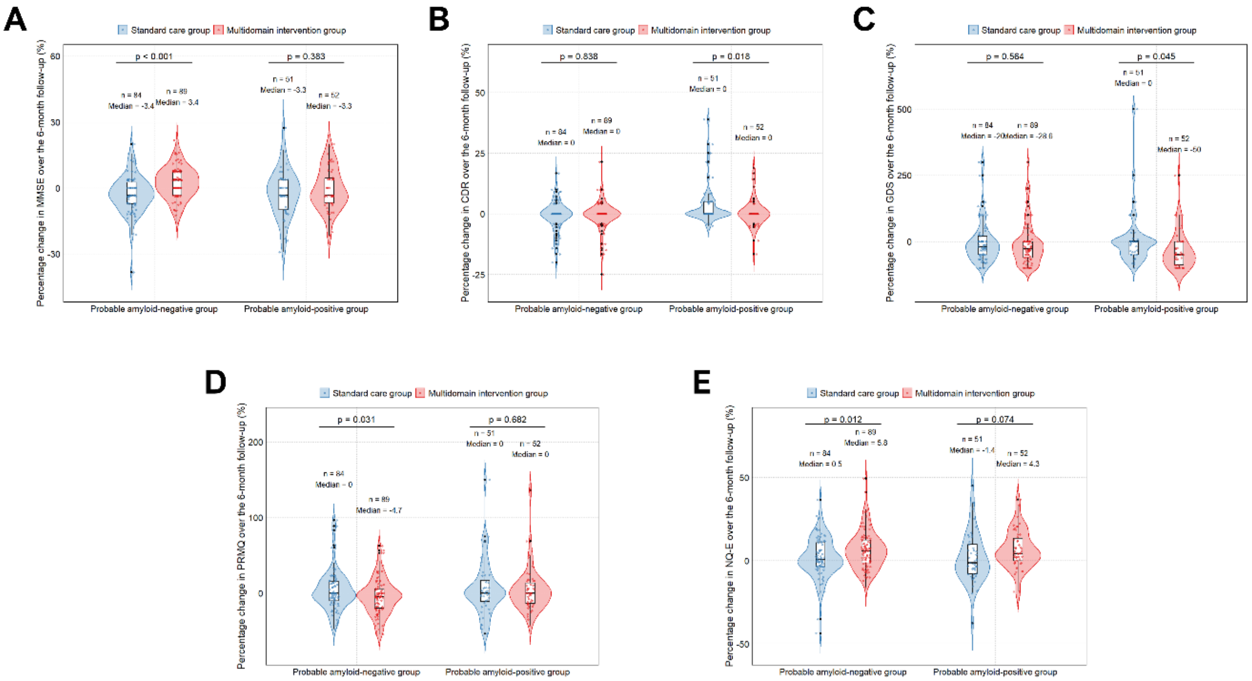

Supplementary Figure 5. Boxplots within violin plots illustrating the effects of multidomain intervention on secondary outcome measures, stratified by probable amyloid-negative and probable amyloid-positive groups. (A) Percentage change in MMSE scores over 6 months. (B) Percentage change in CDR scores over 6 months. (C) Percentage change in SGDS-K scores over 6 months. (D) Percentage change in PRMQ scores over 6 months. (E) Percentage change in NQ-E scores over 6 months.

Abbreviations: CDR, Clinical Dementia Rating; MMSE, Mini-Mental State Examination; NQ-E, Nutrition Quotient for the Elderly; PRMQ, Prospective and Retrospective Memory Questionnaire; RBANS, Repeatable Battery for the Assessment of Neuropsychological Status; SGDS-K, Korean version of the Short Geriatric Depression Scale.

Supplementary Table 1. Six-month changes in cognitive assessment scores, physical activity, and plasma biomarker levels

**Measure**	**Baseline**	**After 6 months**	***p*-value**
Cognitive and physical activity assessments, mean ± SD			
MMSE	26.3 ± 2.3	26.3 ± 2.9	0.750
CDR-SB	1.3 ± 0.9	1.3 ± 1.0	0.984
RBANS index score			

© 2025. Han MH et al. Published online at http://www.aginganddisease.org/EN/10.14336/AD.2025.0645

Total score	87.7 ± 17.1	95.3 ± 18.5	< 0.001
Immediate memory	88.8 ± 14.4	95.8 ± 16.6	< 0.001
Visuospatial capacity	91.1 ± 15.3	94.0 ± 14.6	0.025
Language	97.1 ± 13.4	101.4 ± 14.0	< 0.001
Attention	98.6 ± 14.5	100.2 ± 15.5	0.200
Delayed memory	80.2 ± 18.7	88.8 ± 19.8	< 0.001
Bayer ADL scale	2.3 ± 1.2	2.4 ± 1.4	0.626
SGDS-K	4.8 ± 3.8	3.6 ± 3.5	< 0.001
KQOL scale	33.3 ± 5.3	34.4 ± 5.6	0.019
PRMQ	35.7 ± 11.3	35.8 ± 12.2	0.980
NQ-E	66.0 ± 10.3	68.3 ± 10.1	0.008
MNA	12.0 ± 2.1	12.2 ± 1.9	0.156
PSQI	6.5 ± 4.1	6.2 ± 3.7	0.316
SPPB	9.9 ± 1.9	10.5 ± 1.8	0.001
Blood biomarkers measured with the SIMOA, mean ± SD			
GFAP (pg/mL)	221.7 ± 117.0	228.6 ± 116.7	0.497
BDNF (ng/mL)	14.4 ± 4.5	15.1 ± 4.2	0.046
NfL (pg/mL)	23.6 ± 11.4	29.8 ± 17.0	<0.001
pTau181 (pg/mL)	2.9 ± 1.6	3.1 ±1.7	0.268

Abbreviations: ADL, Activities of Daily Living; BDNF, brain-derived neurotrophic factor; CDR-SB, Clinical Dementia Rating-Sum of Boxes; GFAP, glial fibrillary acidic protein; KQOL, Korean Quality of Life; MNA, Mini Nutritional Assessment; MMSE, Mini-Mental State Examination; NfL, neurofilament light chain; NQ-E, nutrition quotient for the elderly; pTau181, phosphorylated tau at threonine 181; PRMQ, Prospective and Retrospective Memory Questionnaire; PSQI, Pittsburgh Sleep Quality Index; RBANS, Repeatable Battery for the Assessment of Neuropsychological Status; SD, standard deviation; SGDS-K, Short Geriatric Depression Scale-Korean version; SIMOA, Single Molecule Array; SPPB, Short Physical Performance Battery.

© 2025. Han MH et al. Published online at http://www.aginganddisease.org/EN/10.14336/AD.2025.0645

SUPPLEMENTARY DATA

Supplementary Table 2. Sensitivity analysis using Rubin's multiple imputation: Multivariable linear regression analysis of percentage changes in RBANS scores over 6 months, separately including baseline GFAP levels or 6-month GFAP levels as independent variables

Multivariable linear regression analysis				
**All study participants (n = 300)**				
	**Baseline GFAP levels**		**6-month GFAP levels**	
Variable	β (95% CI)	*p*-value	β (95% CI)	*p*-value
Sex (female)	0.897 (-2.13 to 3.92)	0.561	0.864 (-2.15 to 3.88)	0.574
Age (years)	-0.029 (-0.29 to 0.23)	0.829	-0.023 (-0.29 to 0.24)	0.864
Multidomain intervention	**5.516 (2.95 to 8.09)**	**< 0.001**	**5.590 (3.00 to 8.18)**	**< 0.001**
APOE4 positivity	-0.945 (-3.82 to 1.93)	0.518	-1.046 (-3.91 to 1.81)	0.473
GFAP levels	**-0.019 (-0.031 to -0.006)**	**0.003**	**-0.018 (-0.030 to -0.005)**	**0.006**
Family history of dementia	-1.319 (-4.10 to 1.47)	0.353	-1.262 (-4.06 to 1.54)	0.377
Hypertension	0.865 (-1.90 to 3.63)	0.539	0.819 (-1.96 to 3.59)	0.562
Diabetes	-0.246 (-3.33 to 2.84)	0.876	-0.244 (-3.34 to 2.85)	0.877
Hyperlipidemia	-0.887 (-3.55 to 1.78)	0.514	-0.762 (-3.43 to 1.91)	0.575
Hypothyroidism	-0.209 (-7.46 to 7.05)	0.955	-0.546 (-7.84 to 6.75)	0.883
Medication for AD	-2.44 (-5.58 to 0.70)	0.127	-2.662 (-5.78 to 0.46)	0.094

Abbreviations: AD, Alzheimer`s disease; APOE4, apolipoprotein E4; CI, confidence interval; GFAP, glial fibrillary acidic protein; RBANS, Repeatable Battery for the Assessment of Neuropsychological Status

Supplementary Table 3. Demographic and clinical characteristics of the KBASE-V Cohort

**Characteristics**	**Total**
Number	174
Sex, female, n (%)	102 (58.6)
Age, mean ± SD, y	69.77± 8.24
Baseline diagnosis, n (%)	
Cognitive unimpaired	97 (55.7)

© 2025. Han MH et al. Published online at http://www.aginganddisease.org/EN/10.14336/AD.2025.0645

MCI	36 (20.7)
AD	41 (23.6)
Baseline Centiloid, mean ± SD	18.58± 37.10
Amyloid positivity (Centiloid ≥ 37)	43 (24.7)
Baseline pTau-181, mean ± SD, (pg/ml)	2.96 ± 2.31

Abbreviations: AD, Alzheimer`s disease; KBASE-V, Korean Brain Aging Study for the Early Diagnosis and Prediction of Alzheimer's Disease; MCI, mild cognitive impairment; pTau-181, plasma phosphorylated tau at threonine 181; SD, standard deviation.

Supplementary Table 4. Sensitivity analysis using Rubin's multiple imputation: Multivariable linear regression of percentage changes in RBANS total scale index scores at a 6-month follow-up period among participants with available plasma pTau181 data (modified ITT population), classified as probable amyloid-negative or probable amyloid-positive

**Multivariable linear regression analysis**				
**Participants classified as probable amyloid-negative or amyloid-positive (n = 276)**				
	Probable amyloid negative group (n = 173)		Probable amyloid positive group (n = 103)	
Variable	β (95% CI)	*p*-value	β (95% CI)	*p*-value
Sex (female)	-0.006 (-3.97 to 3.95)	0.997	2.236 (-2.84 to 7.31)	0.387
Age (years)	-0.234 (-0.59 to 0.12)	0.193	0.216 (-0.22 to 0.65)	0.331
Multidomain intervention	**3.899 (0.39 to 7.41)**	**0.029**	**5.232 (0.10 to 10.37)**	**0.046**
APOE4 positivity	-0.775 (-5.11 to 3.56)	0.726	-1.513 (-6.14 to 3.12)	0.522
High baseline GFAP group (above median)	-1.901 (-5.94 to 2.14)	0.356	-4.643 (-10.04 to 0.75)	0.092
Family history of dementia	-0.754 (-4.50 to 2.99)	0.693	-0.968 (-5.98 to 4.04)	0.705
Hypertension	0.523 (-3.05 to 4.10)	0.774	0.032 (-4.85 to 4.91)	0.990
Diabetes	-0.603 (-4.82 to 3.61)	0.779	1.831 (-3.38 to 7.05)	0.491
Hyperlipidemia	-0.716 (-4.49 to 3.06)	0.710	0.174 (-4.42 to 4.77)	0.941
Hypothyroidism	-3.152 (-14.30 to 8.00)	0.579	3.455 (-6.36 to 13.27)	0.490

Medication for AD	-1.091 (-5.96 to 3.78)	0.660	-3.983 (-9.22 to 1.26)	0.135

Abbreviations: AD, Alzheimer`s disease; APOE4, apolipoprotein E4; CI, confidence interval; GFAP, glial fibrillary acidic protein; ITT, intention-to-treat; RBANS, Repeatable Battery for the Assessment of Neuropsychological Status.

Supplementary Table 5. Sensitivity analysis using Rubin's multiple imputation: Multivariable linear regression of percentage changes in RBANS total scale index scores at a 6-month follow-up period, stratified by standard care and multidomain intervention groups, among individuals with probable amyloid deposits in the modified ITT population

**Multivariable linear regression analysis**				
**Individuals with probable amyloid deposit (n = 103)**				
	Standard care group (n = 51)		Multidomain intervention group (n = 52)	
Variable	β (95% CI)	*p*-value	β (95% CI)	*p*-value
Sex (female)	3.059 (-5.45 to 11.57)	0.481	1.852 (-4.33 to 8.04)	0.557
Age (years)	-0.139 (-0.79 to 0.51)	0.674	0.444 (-0.20 to 1.09)	0.179
APOE4 positivity	-1.516 (-8.93 to 5.90)	0.688	0.402 (-6.81 to 7.62)	0.913
High baseline GFAP group (above median)	-0.774 (-9.61 to 8.06)	0.863	**-8.574 (-16.66 to -0.49)**	**0.038**
Family history of dementia	-3.469 (-11.61 to 4.67)	0.403	1.063 (-5.86 to 7.98)	0.763
Hypertension	2.906 (-5.21 to 11.02)	0.480	-0.612 (-8.30 to 7.07)	0.876
Diabetes	0.265 (-9.49 to 10.02)	0.957	0.961 (-7.05 to 8.97)	0.813
Hyperlipidemia	-0.269 (-7.48 to 6.94)	0.942	-0.980 (-7.44 to 5.48)	0.766
Hypothyroidism	14.507 (-8.52 to 37.53)	0.217	-1.487 (-12.63 to 9.65)	0.793
Medication for AD	-7.206 (-14.14 to -0.28)	0.042	0.012 (-7.30 to 7.32)	0.997

Abbreviations: AD, Alzheimer`s disease; APOE4, apolipoprotein E4; CI, confidence interval; GFAP, glial fibrillary acidic protein; ITT, intention-to-treat; RBANS, Repeatable Battery for the Assessment of Neuropsychological Status.

© 2025. Han MH et al. Published online at http://www.aginganddisease.org/EN/10.14336/AD.2025.0645

SUPPLEMENTARY DATA

STUDY PROTOCOL

Protocol for: Plasma GFAP and Amyloid Pathology Predict Cognitive Response to Multidomain Interventions in MCI
This trial protocol has been provided by the authors to give readers additional information about the work.

Protocol Synopsis

Study Name	A multicenter randomized controlled study to evaluate the efficacy of a 24-week multidomain intervention via face-to-face and video communication platforms in mild cognitive impairment
Study design	Investigator-initiated, multicenter, randomized controlled studies
Study Period	IRB approval date through June 30, 2023
Number of subjects	300
Research background	In the FINGER study, an active multidomain intervention of exercise, diet, cognitive training, and vascular disease prevention and management in non-demented older adults at high risk for dementia significantly improved cognitive function after 24 months compared to the control group. In the Korean Gold Medal Project study, cognitive function was significantly improved after 18 months in the intervention group that received education about the multidomain lifestyle changes, motivational enhancement, and appropriate rewards for multidomain lifestyle changes compared to the control group.<br>The researchers developed a Korean multidomain intervention program for dementia prevention consisting of cognitive training, exercise, nutrition management, vascular disease risk factor management, and motivation enhancement with the support of the Korea government from May 2018 to December 2020, and conducted a randomized controlled feasibility trial to investigate the applicability of the program, demonstrating that the program was applicable and safe.<br>In this study, we aim to validate the Korean multidomain intervention program for mild cognitive impairment (MCI), that has demonstrated good applicability.
The Purpose	**1. Primary objective**<br>- To investigate the efficacy of a 24-week multidomain intervention consisting of cognitive training, exercise, nutrition, vascular disease risk factor management, and motivation enhancement in MCI. The primary efficacy measure is the change of total scale index score of the Repeatable Battery for the Assessment of Neuropsychological Status (RBANS) from baseline to the study end.<br>**2. Secondary objectives**<br>- Evaluate the effect of the intervention program on overall cognitive function.<br>- Evaluate the effectiveness of the intervention program on improving subjective memory.<br>- Evaluate the effectiveness of the intervention program on improving mood.<br>- Evaluate the effectiveness of the intervention program in improving quality of life<br>- Evaluate the effectiveness of the intervention program in improving daily functioning.

© 2025. Han MH et al. Published online at http://www.aginganddisease.org/EN/10.14336/AD.2025.0645

SUPPLEMENTARY DATA

	- Survey the satisfaction of the intervention program.<br>- Compare progression to dementia between the intervention and control groups.<br>- Evaluate the effectiveness of the intervention program in improving metabolic and vascular risk factors.<br>- Evaluate the effectiveness of the intervention program in improving physical function.<br>- Evaluate the effectiveness of the intervention program in improving eating habits.<br>- Evaluate the effectiveness of the intervention program on motivation.<br>- Investigate the impact of the intervention program on sleep.<br>- Investigate the tolerability (retention rate) of the intervention program.<br>- Investigate adherence to the intervention program.<br>- Investigate the safety of multidomain interventions.<br>**3. Other exploratory objectives**<br>- The effectiveness of the intervention program is investigated by dividing the participants into two groups: amnestic MCI and non-amnestic MCI<br>- The effectiveness of the intervention program is investigated by dividing the participants into two groups: APOE ε4 carriers and noncarriers<br>- The effectiveness of the intervention program is investigated by dividing the participants into two groups: male and female<br>- To investigate changes in neurotrophic factors, neurodegeneration factors, and neuroinflammation associated factors in the blood before and after the intervention program.<br>- To investigate the effect of multidomain interventions according to AD polygenic risk score.<br>- To investigate the relevance of cognitive reserve to multidomain intervention effects.
Inclusion and exclusion criteria	**<Inclusion criteria>**<br>1. Age: 60-85 years old<br>2. Have one or more of the following risk factors for dementia<br>1) Hypertension<br>2) Diabetes Mellitus<br>3) Dyslipidemia<br>4) Obesity: BMI ≥ 25 kg/m^2<br>5) Abdominal obesity: abdominal circumference ≥90cm for men and ≥85cm for women<br>6) Metabolic Syndrome<br>7) Smoking<br>8) Education ≤ 9 years<br>9) Exercising less than the WHO's exercise guidelines for older adults (150 minutes per week of moderate-intensity aerobic activity or 75 minutes per week of vigorous-intensity aerobic activity)<br>10) Lack of social activity<br>3. Participant or informant complains of cognitive decline<br>4. A performance score that is lower than 1.0 standard deviations below the age- and education-adjusted normative means for one or more of the verbal delayed recall, visual delayed recall, executive functioning, naming, visuospatial function, and attention tests.<br>5. No significant functional impairment of activities of daily living.<br>6. Score on the MMSE is greater than or equal to 1.5 standard deviations below the age- and education-adjusted normative means.<br>7. Can use a Tablet PC with training, or has someone who can help them use the Tablet PC.

© 2025. Han MH et al. Published online at http://www.aginganddisease.org/EN/10.14336/AD.2025.0645

	8. Has a reliable informant who knows the participant well enough to provide accurate information about the participant.<br>9. The participant has given written consent to participate in the study.<br>**<Exclusion Criteria>**<br>1. Major psychiatric illness such as major depressive disorders<br>2. Dementia<br>3. Other neurodegenerative disease (e.g., Parkinson's disease)<br>4. Malignancy within five years<br>5. Cardiac stent or revascularization within one year<br>6. Serious or unstable symptomatic cardiovascular disease<br>7. Other serious or unstable medical disease such as acute or severe asthma, active gastric ulcer, severe liver disease, or severe renal disease<br>8. Severe loss of vision, hearing, or communicative disability<br>9. Illiteracy<br>10. Significant laboratory abnormality that may result in cognitive impairment<br>11. Any conditions preventing cooperation as judged by the study physician<br>12. Unable to participate in exercise program safely<br>13. Coincident participation in any other intervention trial
Research methods	- Randomization will be performed using a permuted block randomization technique with stratified for participating centers using the SAS macro program, with 1:1 allocation to the control and intervention groups.<br>**<Intervention Group>**<br>- The intervention arm will receive the multidomain intervention either face-to-face at the site or virtually via ZOOM, a video communication platform on a tablet PC provided by the study.<br>- During the first face-to-face intervention, a guidebook and training on how to use a tablet PC and access ZOOM as well as additional training during each subsequent visit, as necessary, will be provided.<br>1. Cognitive training<br>- Conduct in-person and virtual training using the SUPERBRAIN app's cognitive training programs on tablets.<br>- Training on how to use the tablet PC and access the ZOOM during initial in-person training.<br>- Allow caregivers to assist with at-home lessons in computerized cognitive training. Caregivers can also be trained on how to use ZOOM and tablet PCs so they can support their loved one's in-home interventions.<br>A. 0-8 weeks<br>- Weekly visits for 50 minutes in groups of 3-8 people with qualified health professionals (psychologist, occupational therapist, nurse, etc.) to perform computerized cognitive training on the SUPERBRAIN app on a tablet PC to improve episodic memory, executive function, attention, working memory, calculation, and visuospatial function.<br>- Conduct cognitive training on the SUPERBRAIN app at home once a week and attend one ZOOM class under the guidance of a qualified health professional.<br>B. 9-24 weeks<br>- Computerized cognitive training using the SUPERBRAIN app on a tablet PC under the guidance of a qualified health professional in a group for 50 minutes during a facility visit once every two weeks.

© 2025. Han MH et al. Published online at http://www.aginganddisease.org/EN/10.14336/AD.2025.0645

	- Perform cognitive exercises on their own in the SUPERBRAIN app twice a week when they are not in the clinic and once a week when they are in the clinic, and participate in ZOOM classes taught by a qualified health professional.<br>2. Exercise<br>- 50 minutes 3 times per week<br>- Perform aerobic exercise/strength/balance/flexibility/small muscle exercises under the guidance of a qualified exercise professional.<br>- Participants who were unable to complete the in-person or virtual exercise program at the scheduled time were asked to watch a video recording of each session on a tablet and perform the exercise on their own, with the study coordinator checking for compliance on the admin homepage.<br>A. 0-8 weeks<br>- Come in once a week for a 50-minute workout in a group of 3-8 people. Motivating and addressing challenges at home.<br>- Twice a week for 50 minutes at home with a qualified exercise professional using ZOOM on a tablet PC. Nine floor plates and bands will be provided for home workouts.<br>B. 9-24 weeks<br>- Come in once every two weeks for 60 minutes of exercise in groups of 3-8 people.<br>- Exercised with a qualified exercise professional at home for 50 minutes with ZOOM on a tablet PC three times a week when not in the facility and twice a week when in the facility.<br>3. Vascular risk factor management: face-to-face education on vascular risk factor management by a research nurse using educational materials from the SUPERBRAIN app at baseline, and monitoring of weight, blood pressure, abdominal crimcumference, alcohol consumption, and smoking every 4 weeks. Face-to-face physician counseling at baseline and week 12.<br>4. Nutrition<br>- 3 one-on-one personalized disease education sessions with a dietitian via phone consultation<br>- 12 group in-person sessions with a research nurse using videos from nutrition experts, or 12 individual viewings of nutrition education videos in the SUPERBRAIN app.<br>- Complete the MIND Diet Checklist weekly in the SUPERBRAIN app.<br>5. Motivation: 4 motivational group face-to-face sessions conducted by a trained nurse and weekly self-assessment of dementia prevention activities in the SUPERBRAIN app.<br>**<Controls>**<br>- Physician consultation at baseline and prescription of medications as needed to manage vascular risk factors.<br>- Participants will receive educational booklets on vascular risk factors and a booklet containing lifestyle guidelines for dementia prevention.<br>- Receiving usual care during the study.<br>**<Evaluation>**<br>1. Clinical assessment<br>- Demographic and background information, current/past medical conditions, and concomitant medications<br>- Height, weight, blood pressure, pulse, abdominal circumference, physical examination, neurologic examination<br>- RBANS: Memory/Visuoconstuction/Language/Attention Assessment<br>- MMSE: Global assessment of cognitive function, 0-30<br>- Clinical Dementia Rating (CDR) scale: Assessment of global cognitive functioning

© 2025. Han MH et al. Published online at http://www.aginganddisease.org/EN/10.14336/AD.2025.0645

SUPPLEMENTARY DATA

	- Bayer-ADL (Study Partner Questionnaire): Assessment of Activities of Daily Living (ADL), 1-10<br>- Geriatric Depression Scale-15-item (Participant Questionnaire): assesses depression, 0-15<br>- PRMQ (Participant, Study Partner Questionnaire): subjective memory Impairment questionnaire, 16-80 points<br>- QOL-AD (Participant, Study Partner Questionnaire): Quality of Life Assessment, 0-52 points<br>- SPPB: Balance test, gait speed test, lower extremity muscle strength assessment, 0-12 points<br>- Global Physical Activity Questionnaire (GPAQ)<br>- Fitness measures: 30-second sit-and-stand counts, 2-minute walk in place<br>- Nutrition Quotient for Elderly (Participant Questionnaire): 19 questions about nutrient intake<br>- MNA: Assesses nutrition by examining food intake, weight change, BMI, etc.<br>- Self Determination Index: assesses personality factors, readiness to change, situational motivation, self-efficacy, benefits and barriers, and beliefs about dementia prevention activities<br>- Korean version of the Pittsburgh Sleep Quality Index<br>- Korean version of the Cognitive Reserve Indicator Questionnaire: assessing lifetime educational, occupational, and leisure activities. Administered at baseline only.<br>- Satisfaction questionnaire: administered in the intervention group at weeks 12 and 24<br>2. Blood tests<br>- CBC, HbA1c, AST, ALT, T3, TSH, Free T4, total cholesterol, Triglyceride, HDL-C, LDL-C, Vitamin B12, Folate, 25-Hydroxyvitamin D, urinalysis, RPR precision test, APOE test at the time of screening<br>- HbA1c, triglycerides, HDL-C, LDL-C, total cholesterol, and glucose at 24 weeks<br>3. InBody: body composition analysis, skeletal muscle mass, body fat mass<br>4. Safety assessment: Study coordinators will evaluate the occurrences of adverse events (AEs) when participants visit a facility, when AEs take place, and at the end of the study.<br>5. Adherence: Real-time adherence assessment during face-to-face intervention and non-face-to-face intervention via video communication platform, calculating adherence (%) for each of intervention domains and total adherence (%).<br>6. Tolerability: Investigate the percent retention at the end of the study<br>7. Exploratory investigations: investigating the brain mechanisms of multidomain interventions.<br>- Serum brain derived neurotrophic factor (BDNF), plasma neurofilament light chain as a neurodegenerative protein, plasma glial fibrillary acidic protein (GFAP) as an inflammatory factor, and plasma ptau181 will be measured to compare changes before and after the multidomain intervention program.
Statistical analysis	1. Demographics and background information<br>Background and demographic characteristics are presented using summary statistics. Differences between groups were examined using Student t-tests for continuous variables and chi-squared tests for categorical variables.<br>2. Tolerability<br>It is examined by the retention rate (%) at the end of the study.<br>3. Adherence to multidomain interventions<br>Calculate program participation rates for multidomain interventions to calculate adherence (%) for each intervention area and overall.<br>4. Evaluate the effectiveness of multidomain interventions

© 2025. Han MH et al. Published online at http://www.aginganddisease.org/EN/10.14336/AD.2025.0645

SUPPLEMENTARY DATA

	Changes in the total scale index score and index scores of each cognitive domain of the RBANS will be compared between the intervention and control groups using a linear mixed model with group, visit, group × visit interaction, and the baseline score as fixed effects. Secondary efficacy variables, MMSE, CDR-SB, Bayer-ADL, PRMQ, GDS-15, QOL-AD, SPPB, 30 s sit-to-stand test, 2 min stepping test, NQ-E, MNA, SDI, Pittsburgh Sleep Quality Index, blood pressure, BMI, HbA1c, fasting glucose, LDL-C, HDL-C, total cholesterol, and triglyceride will be compared between control and intervention groups using analysis of covariance (ANCOVA) corrected for baseline scores as covariates.<br>5. Safety<br>Adverse events in the intervention and control groups will be examined using the chi-squared test.<br>6. Exploratory analytics<br>1) Changes in plasma ptau181, serum brain-derived neurotrophic factor, plasma neurofilament light chain, and plasma glial fibrillary acidic protein from baseline to the study endpoint will be compared using analysis of covariance with the baseline score as a covariate.<br>2) Analyze the effect of the multidomain intervention in the same way as in '4. Evaluation of the effectiveness of multidomain interventions' by dividing the APOE ε4 carriers and noncarriers.<br>3) Analyze the effectiveness of multidomain interventions by dividing them into amnestic and nonamnestic MCI in the same way as in '4. Evaluation of the effectiveness of multidomain interventions'.<br>4) Analyze the effect of the multidomain intervention in the same way as in '4. Evaluation of the effectiveness of multidomain interventions' by dividing the men and women.

Table 1. Schedule of procedure/assessments

**Duration**	**Screening**	**Baselines :Randomize**		**Final evaluation**
**Visit**	**V1**	**V2**	**V3**	**V4**
**week**	-56 to -1 d	0 d	12 wks ± 1 wk	24 wks ± 2 wks (Early termination)
Participants written consent	X			
Inclusion/exclusion criteria	X	X		
Demographic and background information	X			
Blood pressure, pulse, weight, abdominal position, alcohol consumption, smoking	X	X	X	X
Height	X			
Physical and neurological exams	X			X
Concomitant medications	X	X	X	X
MMSE, CDR	X			X
K-IADL	X			
RBANS		X	X	X
Bayer-ADL (Research Partner)	X			X
GDS-15 (Participant)		X		X
QOL-AD (Participant, Research Partner)	X			X
PRMQ (Participant, research partner)	X			X

NQ-E (Participant)		X		X
MNA		X		X
Self Determination Index (Participant)		X		X
Pittsburgh Sleep Quality Index (Participant)		X		X
GPAP (Participant)	X			X
Short Physical Performance Battery	X			X
Cognitive Reserve Indicator Questionnaire	X			
InBody Inspection	X			X
30 s sit-to-stand	X			X
2 m stepping test				
Blood tests	X			X
Adherence to intervention programs			X[1]	X[1]
Safety assessment		X	X	X
Satisfaction questionnaire (Participant)			X[1]	X[1]
Clinical Trial Completion Template				X

[1]Intervention group only; MMSE, Mini -Mental State Examination; CDR, Clinical Dementia Rating scale; K-IADL, Korean Instrumental Activities of Daily Living; RBANS, Repeatable Battery for the Assessment of Neuropsychological Status; GDS-15, Geriatric Depression Scale-15 items; QOL-AD, Quality of life-Alzheimer's disease; PRMQ, Prospective and Retrospective Memory Questionnaire; NQ-E, Nutrition Quotient for Elderly; MNA, Mini Nutritional Assessment; GPAP, Global Physical Activity Questionnaire.

Table of Contents

1. Study title 11
2. Principal investigator 11
3. Background 11
4. Study objective 12
4.1. Research hypothesis 12
4.2. Primary objective 12
4.3. Secondary objectives 13
4.4 Other exploratory objectives 14
5. Study subjects 14
5.1. Infication 14
5.2. Target number of subjects 14
5.3. Inclusion Criteria 15
5.4. Exlusion Criteria 16
6. Study methods 17
6.1. Randomization 17
6.2. 24-week multidomain intervention 17
6.3. Education of trainers and assessors..20
6.4. Concomitant Medications 20
7. Study assessments..20
8. Criteria for dropping out of the study..24

© 2025. Han MH et al. Published online at http://www.aginganddisease.org/EN/10.14336/AD.2025.0645

9. Statistical Analysis 25
10. Evaluation and reporting of safety, including adverse effects 27
11. Ethical considerations 29
11.1. Comply with applicable laws and ethical principles 29
11.2. Procedures for obtaining human subject consent 29
11.3. Adherence to the protocol 29
11.4. Monitoring studies 29
11.5. Changes to the protocol 29
11.6. Confidentiality.................... 30
11.7. Insurance and liability 30
11.8. Managing researchers' documents 30
12. References 30

1. Study title

1.1 Study title

A multicenter randomized controlled study to evaluate the efficacy of a 24-week multidomain intervention via face-to-face and video communication platforms in mild cognitive impairment

1.2 Study design

Investigator-initiated, multicenter, randomized controlled studies

2. Principal Investigator

Seong Hye Choi MD, PhD
Department of Neurology, Inha University Hospital
27 Inhang-ro, Jung-gu, Incheon 22332, Korea
Tel: +82-32-890-3659, FAX: +82-32-890-1140, E-mail: seonghye@inha.ac.kr

3. Background

South Korea entered an aging society in 2000 with 7.2% of the population aged 65 and older, and in 2017, the proportion exceeded 14%, making it an elderly society. By 2030, the proportion of the population aged 65 and over is expected to reach 24.3%, making it an ultra-elderly society. Korea's aging index was 68.4% in 2010, with 68 elderly people per 100 young people, but it is expected to rise sharply to 104.1% in 2017, 214% in 2030, and 429% in 2050. The number of people living with dementia worldwide is expected to reach 35.6 million in 2010 and nearly triple to 115.4 million by 2050. In Korea, the prevalence of dementia in each year of the future, estimated based on the age, gender, education, and region of residence standardized dementia prevalence rates based on the 2005 census, is expected to increase to 9.08% in 2012, 9.74% in 2020, 9.61% in 2030, 11.21% in

© 2025. Han MH et al. Published online at http://www.aginganddisease.org/EN/10.14336/AD.2025.0645

2040, and 13.17% in 2050, exceeding 1 million in 2027 and reaching 2.12 million in 2050. The societal cost of dementia exceeds the cost of cancer, heart disease, and stroke combined. In Korea, the total annual medical expenditure for dementia was 810 billion won in 2010, the second highest among geriatric diseases. The per capita medical expenditure for dementia in Korea was 3.1 million won per year, the highest among the five chronic diseases (cerebrovascular 2.04 million won, cardiovascular 1.32 million won). It is estimated that the total national cost of dementia in Korea is 8.7 trillion won per year, doubling every 10 years.

Early detection and aggressive intervention to delay the onset of dementia by two years can reduce the prevalence and severity of dementia by as much as 80% in 20 years [1]. Managing modifiable risk factors for dementia can also reduce the prevalence of dementia by 40% [2]. Modifiable risk factors for dementia include low education, high blood pressure, obesity, hearing loss, brain injury, heavy alcohol use, smoking, depression, lack of exercise, social isolation, air pollution, and diabetes. To maximize the effectiveness of dementia prevention, multidomain interventions that integrate these modifiable risk factors are needed. The FINGER study reported that an active multidomain intervention of exercise, diet, cognitive training, and vascular disease prevention and management in high-risk non-demented elderly people significantly improved cognitive function after 24 months compared to the control group [3]. In the Korean Gold Medal Project study, the intervention group that received education on multidomain lifestyle interventions, motivational interviewing, and adequate rewards significantly improved Mini-Mental State Examination (MMSE) scores after 18 months compared to the control group [4].

It is not possible to apply Western multidomain intervention programs to Korea. The Mediterranean diet has been shown to prevent dementia, but Korean seniors do not consume olive oil, dairy products, or wine. Also, while the elderly in the West tend to exercise at private exercise centers, the elderly in Korea are more likely to exercise outdoors near their homes or participate in public programs. Therefore, it is necessary to localize the program to suit Korean realities.

Supported by the Korea Health Industry Development Institute, we developed a Korean multidomain intervention program consisting of exercise, computerized cognitive training using a tablet PC app (SUPERBRAIN App), nutrition management, social activities, vascular risk factor management, and motivation enhancement programs from May 2018 to December 2020, and conducted a feasibility RCT to investigate its applicability [5]. In the Feasibility RCT, the retention rate of the 24-week intervention was about 90%, and adherence to the intervention program was excellent at over 90%. We also observed significant improvements in cognitive function, depression, and quality of life in the intervention group compared to the control group, and observed that the developed program was safe [6].

In this study, we aim to conduct a randomized controlled trial to investigate an efficacy of the Korean multidomain intervention program on cognitive function in mild cognitive impairment (MCI),

© 2025. Han MH et al. Published online at http://www.aginganddisease.org/EN/10.14336/AD.2025.0645

SUPPLEMENTARY DATA

4. Study objectives

4.1. Research hypothesis

There will be a difference in cognitive function change from baseline to 24 weeks between the control group and the intervention group receiving a 24-week multidomain intervention delivered via face-to-face and video communication platforms in MCI.

4.2. Primary objective

- To investigate the efficacy of a 24-week multidomain intervention consisting of cognitive training, exercise, nutrition, vascular disease risk factor management, and motivation enhancement in MCI. The primary efficacy measure is the change of total scale index score of the Repeatable Battery for the Assessment of Neuropsychological Status (RBANS) from baseline to the study end.

4.3. Secondary objectives

4.4.1. Evaluate the effect of multidomain interventions on overall cognitive functioning.

4.4.2. Evaluate the effectiveness of multidomain interventions on improving subjective memory.

4.4.3. Evaluate the effectiveness of multidomain interventions on improving mood.

4.4.4. Evaluate the effectiveness of multidomain interventions on improving quality of life

4.4.5. Evaluate the effectiveness of multidomain interventions in improving daily living functioning.

4.4.6. Investigate satisfaction with multidomain interventions.

4.4.7. Compare progression to dementia between the intervention and control groups.

4.4.8. Evaluate the effectiveness of the intervention program in improving metabolic and vascular risk factors.

4.4.9. Evaluate the effectiveness of an intervention program in improving physical function.

4.4.10. Evaluate the effectiveness of the intervention program in improving dietary habits.

4.4.11. Evaluate the effectiveness of intervention programs on motivation.

4.4.12. Investigate the impact of multidomain interventions on sleep.

4.4.13. Investigate the retention rate (tolerability) of multidomain interventions.

4.4.14. Investigate adherence to the intervention program.

4.4 15. Investigate the safety of multidomain interventions.

<Secondary outcome measures>

1) Mini-Mental State Examination (MMSE)

2) Clinical Dementia Raring scale-Sum of Boxes (CDR-SB)

3) Prospective and Retrospective Memory Questionnaire (PRMQ)

4) Geriatric Depression Scale-15 items (GDS-15)

5) Quality of life-Alzheimer's disease (QOL-AD)

6) Bayer Activities of Daily Living (ADL)

© 2025. Han MH et al. Published online at http://www.aginganddisease.org/EN/10.14336/AD.2025.0645

7) Nutrition Quotient for elderly (NQ-E)

8) Mini Nutritional Assessment (MNI)

9) Short Physical Performance Battery (SPPB)

10) 30 s sit-to-stand

11) 2 m stepping test

12) Body Mass Index (BMI), lipid panel. HbA1c, fasting glucose, abdominal circumference, blood pressure

13) Global Physical Activity Questionnaire (GPAQ)

14) Self Determination Index (SDI)

15) Pittsburgh Sleep Quality Index

16) Progression to dementia

17) Satisfaction questionnaire

4.4. Other exploratory objectives

4.4.1. Investigate the effectiveness of the intervention program in two groups: amnestic MCI and nonamnestic MCI.

4.4.2. Investigate the effectiveness of the intervention program in two groups: APOE ε4 carriers and noncarriers

: APOE ε4 is a risk factor for Alzheimer's disease. Since prevention of AD is ultimately important, we would like to further analyze the effectiveness of the intervention program in subjects at high risk for AD.

4.4.3. Investigate the effectiveness of the intervention program in two groups: men and women

4.4.4. To investigate the mechanisms by which multidomain interventions act on the brain, we will examine changes in Brain Derived Neurotrophic Factor (BDNF), neurofilament light chain, Glial fibrillary aci dic protein (GFAP), and pTau181 in the blood before and after multidomain interventions.

4.4.5. To investigate the effect of multidomain interventions according to AD polygenic risk score.

4.4.6. To investigate the relevance of cognitive reserve to multidomain intervention effects.

-

5. Study subjects

5.1. Indication

MCI

5.2. Target number of subjects

In our previous study, the difference in RBANS total scale index score between the control and home-based multidomain intervention groups was 6.2 [6]. The standard deviation of the RBANS total scale index score

© 2025. Han MH et al. Published online at http://www.aginganddisease.org/EN/10.14336/AD.2025.0645

was 19.8 in the control group. The autocorrelation of the RBANS total scale index scores between the baseline and study end was 0.8 in the control group. In this trial, three evaluations of the RBANS are conducted. To achieve a power of 0.8 for detecting a significant difference (P=0.05, two-sided) using the time-averaged difference of repeated measures in the power analysis and sample size program, PASS 11 (NCSS, Kaysville, UT, USA), 134 participants in each study group is required. Anticipating a dropout rate of 10.5% according to our previous report [6], the estimated sample size is 300, with 150 participants per group.

5.3. Inclusion Criteria

1. Age: 60-85 years old
2. Have 1 or more of the following modifiable risk factors for dementia [2, 7]

1) Hypertension: systolic blood pressure ≥140 mmHg or diastolic blood pressure ≥90 mmHg according to the diagnostic criteria of the WHO-International Society of Hypertension (1999) [8] or taking antihypertensive medication

2) Diabetes: 8-hour fasting blood glucose ≥126 mg/dl or symptoms of diabetes (eg, polyuria, weight loss) with a usual blood glucose ≥200 mg/dl or HbA1c ≥ 6.5% or being treated with insulin or oral hypoglycemic agents [9].

3) Dyslipidemia: Total cholesterol ≥ 200 mg/dl or LDL-C ≥130 mg/dl or HDL-C <40 mg/dL or triglycerides ≥150 mg/dL according to the criteria of the National Cholesterol Education Program (NCEP) Expert Panel (2002) [10] or taking medication to lower blood lipids.

4) Obesity: Body Mass Index (BMI) ≥ 25 kg/m^2 according to Asia-Pacific guidelines [11].

5) Abdominal obesity: according to Korean criteria for abdominal circumference [12], men ≥90 cm, women ≥85 cm

6) Metabolic syndrome: Three or more of the following, using Asian criteria [13].

(1) Abdominal circumference: Men ≥ 90 cm, Women ≥ 85 cm

(2) Fasting blood glucose ≥ 100 mg/dl or taking diabetes medication

(3) Blood pressure ≥130/85 mm Hg or taking antihypertensive medication

(4) triglycerides ≥150 mg/dl or taking triglyceride medication

(5) HDL-C: Men < 40 mg/dl, women < 50 mg/dl, or Taking medications for dyslipidemia

7) Smoking: Based on the Centers for Disease Control and Prevention (CDC) definition of a smoker, you have smoked at least 100 cigarettes in your lifetime and have smoked at least one cigarette in the past month [14].

8) Education ≤ 9 years

© 2025. Han MH et al. Published online at http://www.aginganddisease.org/EN/10.14336/AD.2025.0645

9) According to the World Health Organization's (WHO) exercise guidelines for older adults [15], you should get at least 150 minutes of moderate-intensity aerobic activity per week or 75 minutes of vigorous-intensity aerobic activity per week, but if you do not get enough aerobic activity.

10) Lack of social activities: Participating less than twice a week in activities such as social groups (family reunions, alumni associations, hometown associations, employee associations, senior centers, etc.), cultural activity groups (choirs, drawing, theater, movie watching, etc.), sports leisure groups (mountain clubs, early soccer clubs, etc.), volunteer activities, and learning groups (senior colleges, senior classes, educational programs at welfare centers/resident centers/religious institutions), etc.

3. subject or study partner complains of cognitive decline
4. A performance score that is lower than 1.0 standard deviations below the age- and education-adjusted normative means for one or more of the verbal delayed recall, visual delayed recall, executive functioning, naming, visuospatial function, and attention tests from the detailed cognitive functioning test.
5. No significant impairment in activities of daily living.
   : Score on the Korean-Instrumental Activities of Daily Living is less than 0.4 [18].
6. Score on the MMSE is greater than or equal to 1.5 standard deviations below the age- and education-adjusted normative means [19].
7. Can use a Tablet PC with training, or has someone who can help them use the Tablet PC.
8. Have a reliable informant who knows the participant well enough to provide accurate information about the participant.
9. The participant should give written consent to participate in the study.

5.4. Exclusion Criteria

1. Psychiatric illness, such as major depression, is present
2. Dementia
3. Other degenerative brain diseases, such as Parkinson's disease
4. Malignancy within 5 years that has not been declared curable
5. Revascularization or stenting procedure within 1 year
6. Severe or unstable symptomatic cardiovascular disease
7. Evidence of other serious or unstable physical illness, such as acute and severe asthma, active peptic ulcer disease, severe liver disease or renal disease sufficient to require dialysis, or any other medical condition that would interfere with completion of the study
8. Severe vision impairment, severe hearing impairment, or severe deafness that prevents intervention and efficacy evaluation. Communication disorders

© 2025. Han MH et al. Published online at http://www.aginganddisease.org/EN/10.14336/AD.2025.0645

9. Non-literate
10. In the clinician's judgment, there are abnormalities observed on clinical examination that contribute to cognitive decline (e.g., significant thyroid dysfunction, vitamin B12 or folate deficiency, neurosyphilis, etc.)
11. In the judgment of the researcher, is unable to fully and cooperatively participate in the study.
12. Has difficulty safely participating in intervention programs such as exercise, as determined by the researcher
13. Participating in another intervention study

6. Study methods

6.1. Randomization

Randomization will be performed using a permuted block randomization technique with stratified for participating centers using the SAS macro program, with 1:1 allocation to the control and intervention groups. The efficacy assessor will be blinded to the assigned arm.

6.2. 24-week multidomain intervention

6.2.1. Intervention group

- The intervention group will receive face-to-face interventions in a group and individually at a facility, as well as virtual interventions at home via the video communication platform ZOOM on a tablet PC.
- During the first face-to-face intervention, a guidebook and training on how to use a tablet PC and access ZOOM as well as additional training during each subsequent visit, as necessary, will be provided.
- LTE service will be supported if a participant doesn't have Wi-Fi at home.

1) Cognitive training

- Cognitive training is conducted at an institution and at home using the SUPERBRAIN app on a tablet PC provided by the study.
- Training on how to use the tablet PC and access the ZOOM during initial in-person training.
- Caregivers can assist with at-home lessons in computerized cognitive training. Caregivers will also be trained on how to use ZOOM and tablet PCs so that they can support the participant's at-home intervention.

A. 0-8 weeks

- Participants come to an institution once a week to perform computerized cognitive training to improve episodic memory, executive function, attention, working memory, calculation, and visuospatial function using the SUPERBRAIN app on a tablet PC for 50 minutes in a group of 3-8 people under the guidance of a qualified health professional (psychologist, occupational therapist, nurse, etc.); learn how to implement the cognitive training program using a tablet PC; and motivate, and identify and address difficulties in implementing the program at home.
- Once a week, participants practice cognitive training in the SUPERBRAIN app on the tablet PC at home. Participants will engage in a weekly online cognitive training session focused on homework, facilitated by a qualified health

© 2025. Han MH et al. Published online at http://www.aginganddisease.org/EN/10.14336/AD.2025.0645

professional through the ZOOM platform. Implementation of cognitive training in the SUPERBRAIN app will be monitored on the admin homepage.

B. 9-24 weeks

- Participants come to an institution once every two weeks to perform computerized cognitive training using the SUPERBRAIN app on a tablet PC for 50 minutes in a group under the guidance of a qualified health professional. Difficulties when practicing at home will be identified and solved and participants will be motivated.
  - In weeks that include a group session, participants participate in weekly self-administered cognitive training sessions at home. Additionally, they attend a weekly online cognitive training session focused on homework, led by a qualified health professional through the ZOOM platform. During weeks without group sessions, participants will undergo twice-weekly self-administered cognitive training sessions at home. Additionally, Participants will participate in twice-weekly online cognitive training sessions focused on homework and facilitated by a qualified health professional through the ZOOM platform.

2) Exercise

- 50 minutes 3 times per week
- Qualified exercise professionals will lead the exercise program consisted of aerobic exercise, muscle-strengthening exercises targeting major muscle groups, balance, flexibility, and finger and toe exercises, at a facility and via the ZOOM platform.
- It utilizes portable equipment like elastic bands, nine floor plates labeled with numbers, and chairs.
- In-person, group exercises are performed in one room, and a qualified exercise professional stands in the center of the group and leads the exercises, correcting participants' movements as needed. During an in-person exercise session, the qualified exercise professional will pay more attention to and correct problematic movements from each participant's virtual exercise session, and participants will have time to ask the qualified exercise professional additional questions after the session.
- Exercise intensity will be increased at 2-month intervals, assessed by heart rate data obtained by wearing a wearable activity tracker (Fitbit Charge2) during in-person exercise sessions during the first week of the new exercise intensity (Inha University, Ewha Womans University Hospital, and Ajou University Hospital only).
- Participants will be pre-trained to troubleshoot any issues with the videoconferencing program during the virtual exercise program by phone with the study coordinator at each center.
- Participants who were unable to complete the in-person or virtual exercise program at the scheduled time were able to view the recorded video of each session on a tablet PC and perform the exercises themselves, with the study coordinator checking for compliance on the admin homepage.
- Feedback for any problem associated with exercise program will be provided by a community consisted of athletic therapists and researchers.

A. 0-8 weeks

© 2025. Han MH et al. Published online at http://www.aginganddisease.org/EN/10.14336/AD.2025.0645

- Participants will come in a facility once a week for a 50-minute workout in a group of 3-8 people. Participants will be motivated and addressed challenges for at-home exercises.
- Twice a week, 60-minute workouts at home with an athletic trainer via ZOOM, a video communication platform on a tablet PC. Mats and bands will be provided for home workouts.

B. 9-24 weeks

- Participants will participate in group exercise sessions at a facility fortnightly. In weeks containing group sessions, participants will engage in twice-weekly online exercise sessions at home through the ZOOM platform. During weeks without group sessions, participants will participate in thrice weekly online exercise sessions at home using the ZOOM platform.

3) Nutrition

- 12 additional viewing of a nutrition education video created by a nutrition professor on the tablet PC when visiting a facility once every two weeks
  : Nutrition education videos will provide dietary education, practical exercises for facilitating eating changes, and advice on how to cook meals with recommended ingredients through cooking lessons
- Three individual phone consultations (each lasting 30 minutes) by a research dietitian
  : The individual phone consultations are held in the 2nd, 4th, and 8th weeks, and include tailoring the participant's daily dietary needs and educating them on customized diets to manage individual vascular risk factors.
- Participants find motivation to complete the MIND diet [20] checklist weekly using the tablet PC.

4) Vascular and metabolic disease prevention and management

- Before the intervention, the metabolic and vascular risk factors will assessed through blood tests and anthropometric measurements (weight, blood pressure, and waist circumference).
- Hypertension, DM, dyslipidemia, obesity, abdominal obesity, smoking, and heavy alcohol consumption are monitored and managed.
- Each participant meets a study doctor at baseline and week 12. Study doctors will inform participants of their risk factors and prescribe medication if necessary.
- At baseline, participants will be educated by a study nurse using educational materials about their risk factors and lifestyle guidelines for dementia prevention, which will be loaded on a tablet PC.
- They will also meet the study nurse every four weeks for anthropometric measurements and monitoring of smoking and alcohol intake. Measurements will be recorded on the participant's tablet PC application at each visit to help motivate them to change.
- If a participant's risk factors do not improve, the study nurse will re-educate the participant at week 12 using educational materials on the tablet PC.

5) Motivation enhancement

SUPPLEMENTARY DATA

- Motivational enhancement will comprise four group counseling sessions, each lasting 50 minutes, led by a study coordinator in weeks 1, 2, 12, and 24. The motivational enhancement program will aim to instigate, sustain, and reinforce motivation, serving as a psychological resource to support the continuity of dementia prevention activities. The participants' motivation and self-efficacy levels will be evaluated during each session of motivational enhancement.
- Through the family coach program, a family member can reinforce a participant's motivation. Participants will receive cheering video messages from their families or research staff and self-assessments of achievements in the form of pop-up notifications every week before the tablet-based cognitive intervention.

6.2.2. Control Group

- Participants in the control group will have a physician consultation at baseline and prescription of medications as needed to manage vascular risk factors.
- Participants will be given educational booklets on vascular risk factors and a booklet containing lifestyle guidelines for dementia prevention.
- Participants will be received usual care during the study
- Participants in the control group will be notified that they can participate in the intervention after the study ends.

6.3 . Education of trainers and assessors

- Workshops will be conducted to educate qualified exercise professionals, qualified health professionals , and research coordinators at each site about the multidomain intervention program, research methods, scheduling, and E-CRF completion.
- Manuals and videos will be distributed to ensure standardized interventions.
- Workshops will be conducted to train outcome assessors at each study site.
- The research coordinators will be based at Inha University Hospital, qualified health professionals will be based at Ewha Womans University Hospital, and qualified exercise professionals will be based at Aj ou University Hospital, and will conduct quality control by operating chat rooms and regular meetings t hroughout the study period.

6.4. Concomitant Medications

- Participants are eligible if they have been on a stable acetylcholinesterase inhibitor dose without chang e for at least 8 weeks prior to baseline assessment, or if they are not using these medications. They will not change their use or dose of these medications until the end of the 24-week study.
- Antidepressants and anti-anxiety medications that have been taken at a stable dose for at least 8 weeks prior to baseline assessments will be continued throughout the 24-week study with no change in dose. I f you wish to discontinue prior to study entry, do so 2 weeks prior to baseline assessment.
-

© 2025. Han MH et al. Published online at http://www.aginganddisease.org/EN/10.14336/AD.2025.0645

SUPPLEMENTARY DATA

7. Study assessments

Table 1 lists all endpoints, with an 'X' marking the visit at which the assessment will occur. Participants will attend all visits on the assigned day or within 14 days, 7 days before and 7 days after the assigned day.

Participants who drop out of the study early after baseline for any reason will be rescheduled for a visit as soon as possible to perform all assessments scheduled for the final visit.

- ***Screening*** visit: Informed consent, study partner consent, inclusion/exclusion criteria, demographics and background information, current/past medical history, concomitant medications, vital signs, height, weight, abdominal circumference, physical examination, neurologic examination, MMSE, CDR, K-IADL, SPPB, Global Physical Activity Questionnaire, Bayer ADL, PRMQ, QOL-AD, Korean version of the Cognitive Prospective Indicators Questionnaire, blood tests, InBody, 30 s sit-to-stand, and 2 m stepping test.
- ***Baseline*** visit: Inclusion/exclusion criteria, blood pressure, pulse, weight, abdominal circumference, alcohol and tobacco use, concomitant medications, RBANS, GDS-15, SDI, NQ-E, MNA, Korean version of the Pittsburgh Sleep Quality Index.
- ***12-week visit:*** Blood pressure, pulse, weight, abdominal circumference, alcohol and tobacco use, concomitant medications, RBANS, satisfaction questionnaire (intervention arm), adherence to the multidomain intervention program, and safety.
- **Final evaluation (*within 2 weeks of the end of the* 24-week multidomain intervention program*) and an assessment visit at early termination*:** Blood pressure, pulse rate, weight, abdominal circumference, alcohol and tobacco use, concomitant medications, MMSE, CDR, RBANS, Bayer ADL, GDS-15, QOL-AD, PRMQ, SPPB, physical examination, 30 s sit-to-stand, 2 m stepping test, Global Physical Activity Questionnaire, NQ-E, MNA, SDI, Korean version of Pittsburgh Sleep Quality Index, blood tests, adherence to the multidomain intervention program, and safety. Satisfaction questionnaires will be administered in the intervention arm only.

***Unscheduled visits*.** Perform an assessment related to the cause of the unscheduled visit.

1) RBANS

Memory tests include learning word lists and recalling stories. Visuoconstruction tests include drawing shapes and tracing lines. Language tests consist of picture naming and semantic fluency. Attention tests consist of digit span and digit symbol. It takes about 30 minutes, and higher scores indicate good cognitive functioning. It will be assessed at baseline, Week 12, and Week 24.

2) MMSE

It is consisted of time and place, 3-word memory, 100-7 in a row, overlapping pentagons, following

© 2025. Han MH et al. Published online at http://www.aginganddisease.org/EN/10.14336/AD.2025.0645

commands, reading, writing, repeating, and naming (0-30 points). It reflects overall cognitive function. Higher scores indicate better cognitive functioning. Administered at screening and final evaluation.

3) CDR scale

It reflects overall cognitive functioning and assesses six domains: memory, attention, judgment and problem solving, social activities, home life and hobbies, hygiene and grooming. The Global CDR (0-3 points) and CDR-SB (0-18 points) are calculated by summing the scores of all six items. Higher scores indicate poorer cognitive function. Administered at screening and final evaluation.

4) Geriatric Depression Scale-15 items (GDS-15)

Participants mark "yes" or "no" as they read a questionnaire about 15 depression symptoms. Scores range from 0-15, with higher scores indicating depression. In the Korean validation study, a score of 8 or higher was considered suspicious for depression. Administered at baseline and final evaluation.

5) Bayer ADL (Research Partner Questionnaire)

It consists of 25 items, which are read by the caregiver and checked about the participant. Each item is scored on a 1-10 point scale. Higher scores indicate greater functional impairment. Administered at baseline and final evaluation.

6) QOL-AD (Participant, Research Partner Questionnaire)

It asks the subject and ask the caregiver about the subject's quality of life. It has a score distribution of 0-52 points. Higher scores indicate better quality of life. Administered at baseline and final evaluation.

7) PRMQ (Participant, Research Partner Questionnaire)

It consists of 16 questions. Eight items are about prospective memories and eight are about retrospective memories. Each item is scored on a 1-5 scale, so the total score can range from 16-80 points. Higher scores indicate more severe memory impairment. Administered at baseline and final evaluation.

8) K-IADL

It is an 11-item screening tool that assesses instrumental activities of daily living (0-3 points). A score of 0.4 or higher suggests dementia. Administered at screening.

9) Short Physical Performance Battery (SPPB)

It consists of chair stand-up test with balance test, gait speed test, and lower extremity muscle strength assessment (0-12 points). Higher scores indicate better physical function. Performed at screening and final evaluation.

10) Global Physical Activity Questionnaire (GPAQ)

A questionnaire that examines the extent to which a person engages in work-related activities, traveling from place to place, leisure activities, and sedentary activities during a typical week, divided into moderate and vigorous activities. Administered at screening and final evaluation.

11) Physical fitness test

© 2025. Han MH et al. Published online at http://www.aginganddisease.org/EN/10.14336/AD.2025.0645

Measures the number of times a person sits up from a chair for 30 seconds and walks in place for 2 minutes, administered at screening and final evaluation.

12) InBody Inspection

Body composition analysis, skeletal muscle mass, body fat mass. Performed at screening and final evaluation.

13) NQ-E (participant)

It consists of 19 questions about usual nutrient intake. Participants mark as they read. Higher scores indicate better nutritional status. Administered at screening and final evaluation.

14) Mini Nutritional Assessment

It assesses nutrition by examining weight change, BMI, and more (0-14 points). Higher scores indicate better nutritional status. Administered at screening and final evaluation.

15) SDI

It assesses personality five-factor scale, readiness to change, situational motivation, self-efficacy, benefits and barriers, and beliefs about dementia prevention activities. Administered at baseline and final evaluation.

16) Korean version of Pittsburgh Sleep Quality Index

It is composed of seven subscales: Subjective sleep quality, sleep latency, sleep duration, usual sleep efficiency, sleep interruptions, sleeping pills use, and daytime activity impairment. Each of the seven subscales is scored on a scale of 0-3 for each criterion, with a minimum score of 0 and a maximum score of 21. A total score of 8 or less indicates good sleep quality and a score of 9 or more indicates poor sleep quality. Administered at screening and final evaluation.

17) Korean version of the Cognitive Reserve Index Questionnaire (CRIq)

A questionnaire that measures cognitive reserve by assessing lifetime educational, occupational, and leisure activities. It yields a Total CRIq score, with a mean of 100 and a standard deviation of 15. Administered once at baseline.

18) Satisfaction questionnaire

Examine satisfaction with the program in the intervention group at weeks 12 and 24.

19) Blood tests

- - Blood will be drawn twice: 26.5 cc at screening and 24.5 cc at the end of the study.

A. Examination for health assessment

- - At the time of screening, 8 cc will be delivered to Seoul Clinical Laboratories (SCL) for testing. 3cc of blood will be collected in 1 EDTA tube, shaked and refrigerated (5-8 degrees). Collect 5cc of blood in 1 SST tube, leave at room temperature for 30 minutes, centrifuge (3000 rpm, 10 minutes), and refrigerate (5-8 degrees). Urine should be collected in a tube of 8cc or more and refrigerated (5-8 degrees). CBC, HbA1c, glucose, AST, ALT, BUN, creatinine, T3, TSH, Free T4, total cholesterol, triglyceride, HDL-C, LDL-C,

© 2025. Han MH et al. Published online at http://www.aginganddisease.org/EN/10.14336/AD.2025.0645

Vitamin B12, Folate, 25-Hydroxyvitamin D, urinalysis, RPR, and APOE genotype will be evaluated at SCL . Do not repeat APOE genotyping if previously performed. Do not repeat T3, TSH, and FreeT4 tests if they were performed within 1 year of screening.

- - At the end of the study, 6 cc of blood will be sent to the SCL to test for HbA1c, triglycerides, HDL-C, LDL-C, total cholesterol, and glucose.
- - After testing at the SCL, any remaining samples are immediately discarded.

B. Exploratory research

- Draw 5 ml and 3.5 ml of blood into two SST tubes for serum analysis and 10 cc into an EDTA tube for plasma analysis. Performed at screening and endpoint assessments.

- Serum: Blood in SST tubes is stored upright and allowed to clot for 30 minutes at room temperature before centrifugation at 3000 rpm for 15 minutes at room temperature. All steps up to centrifugation are performed within 1 hour of blood sample collection. Serum from the supernatant in the SCL was aliquoted into 1.5 ml e-tubes in 500 uL increments using a transfer pipette and stored in a freezer below -70 degrees Celsius immediately after aliquoting.

- Plasma: Blood in 10cc EDTA tube, gently invert the tube 5-6 times to mix well, send to SCL, centrifuge at 3000 rpm for 10 minutes at room temperature in SCL, plasma obtained from the upper layer is transferred to 1.5ml e-tube in 500ul increments using a transfer pipette. Immediately stored in a freezer below -70 degrees.

- Remove the plasma from the SCL, separate the remaining Buffy coat, transfer to a 1.5 ml e-tube, and freeze. This will be utilized for APOE polymorphism analysis and AD polygenic risk score calculation. SNP analysis for AD polygenic risk score will be performed at DNALINK utilizing the Axiom® Korean chip.

- Serum BDNF as a neurotrophic factor, plasma neurofilament light chain as a neurodegeneration protein, plasma GFAP as an inflammatory factor, and plasma ptau181 will be tested before and after the multidomain intervention in collaboration with Prof. Seong-Ho Koh of Hanyang University. The blood sent to Hanyang University will be preserved and disposed of after the above tests only until the period specified in the Participants' consent form.

8. Criteria for dropping out of the study

If the investigator determines that continued participation in the study would pose a significant safety risk to the participant, the participant should be withdrawn from the study. A participant will be withdrawn from the study in the following circumstances, as determined by the investigator

- The researcher determines that continued participation in the study would be detrimental to the subject's welfare.

© 2025. Han MH et al. Published online at http://www.aginganddisease.org/EN/10.14336/AD.2025.0645

- Withdrawing consent by a participant
- If at any time, for any reason, the researcher determines that withdrawal is in the best interest of the participant

Violations of the protocol will not result in dropping a participant from the study unless they constitute a significant risk to the safety of the participant. Participants may voluntarily stop participating in the study at any time and for any reason. A participant may be considered to have dropped out if he or she indicates a desire to discontinue participation, fails to return for a visit, or is lost to follow-up for any other reason.

If during the course of the study it is determined that a participant has progressed to dementia, training or follow-up will continue until week 24 if the participant does not refuse. This will not be a criterion for dropping out of the study. However, if acetylcholinesterase inhibitors or memantine are added or changed midway through the study, this should be noted in the concomitant medication section of the case record.

If early withdrawal occurs, the researcher will assess the primary reason for the subject's early withdrawal from the study and document that information in the Study Completion field of the case record. For subjects whose current status is unclear because they have not indicated a desire to discontinue participation and have not attended visits, the researcher will conduct a phone call with the subject to determine the primary reason for the dropout.

9. Statistical Analysis

9.1. Analysis Populations

The endpoints will be evaluated within a modified intention-to-treat population, encompassing all randomized participants who undergo a baseline assessment and at least one post-baseline evaluation and engage in the intervention program at least once if assigned to the intervention group. Additional analyses on per-protocol populations will also be performed.

9.2. Demographics and Background Information

Background and demographic characteristics will be presented using summary statistics.

Summary statistics will include frequencies and percentages for categorical variables, and number of observations (n), mean, standard deviation, minimum, and maximum for continuous variables. Differences in demographic and background information between groups will be examined using student t-tests for continuous variables and chi-square tests for categorical variables.

9.3. Tolerability

The tolerability of the multidomain intervention is examined by the retention rate (%) at the end of the study. The retention rate between the two groups will be compared using chi-square.

9.4. Adherence to Multidomain Interventions

© 2025. Han MH et al. Published online at http://www.aginganddisease.org/EN/10.14336/AD.2025.0645

The adherence rate (%) will be calculated by adding the number of sessions completed and dividing by the number of interventions assigned to each intervention component. The total adherence rate (%) will be calculated by adding the number of sessions completed across all intervention components and dividing by the total number of interventions assigned without modifying the weight of each intervention component.

9.5. Evaluating the Efficacy of Multidomain Interventions

Changes in the RBANS total scale index score will be compared between the intervention and control groups using a linear mixed model with group, visit, group × visit interaction, and the baseline score as fixed effects. Changes in index scores of each cognitive domain of the RBANS will be compared between the intervention and control groups using linear mixed models with group, visit, group × visit interaction, and the baseline score as fixed effects.

Analysis of covariance with a baseline score as a covariate will be used to compare changes from baseline to the study endpoint in the secondary outcomes such as MMSE, CDR-SB, Bayer-ADL, PRMQ, GDS-15, QOL-AD, SPPB, 30 s sit-to-stand test, 2 min stepping test, NQ-E, MNA, SDI, Pittsburgh Sleep Quality Index, blood pressure, BMI, HbA1c, fasting glucose, LDL-C, HDL-C, total cholesterol, and triglyceride.

9.6. Evaluating the Safety of Multidomain Interventions

The safety analysis will be performed on participants who undergo at least one safety evaluation post-baseline and participate in the intervention program at least once if assigned to the intervention group. The chi-square test will compare the incidence of adverse events between the intervention and control groups.

9.7. Exploratory Analysis

1) Changes in plasma ptau181, serum brain-derived neurotrophic factor, plasma neurofilament light chain, and plasma glial fibrillary acidic protein from baseline to the study endpoint will be compared using analysis of covariance with the baseline score as a covariate.

2) The effectiveness of the multidomain intervention will be assessed by dividing the APOE ε4 carriers and noncarriers, as described above (1.6 Evaluating the efficacy of Multidomain Interventions).

3) The effectiveness of the multidomain intervention will be assessed by dividing them into amnestic mild cognitive impairment (MCI) and nonamnestic MCI using the same method as described above (1.6 Evaluating the efficacy of Multidomain Interventions).

4) The effectiveness of the multidomain intervention will be assessed by dividing them into men and women as described above (1.6 Evaluating the efficacy of Multidomain Interventions).

10. Evaluation and reporting of safety, including adverse effects

10.1. Evaluation Methodology

1) An adverse event is any unintended symptom, symptomatology, or illness that was not observed prior to study entry that develops during the study, regardless of causal relationship to the multidomain intervention.

2) If determined to be an adverse event, the symptoms and signs, including onset date and duration, should be fully documented in the adverse event report.
3) In principle, the evaluation of the severity of adverse reactions should be evaluated by the investigator according to the severity of the symptoms in stages, referring to the evaluation criteria.
4) The causal relationship with multidomain interventions shall be evaluated by the physician in charge according to the following classification.
Categorizing causality
Each AE and its relevance to the intervention will be recorded in the CRF by answering the following questions
*Is there a reasonable likelihood that the intervention will result in an AE?
-Yes (Related) There is a reasonable probability of causation between the intervention and the AE.
-Not Related (Not Related) No reasonable possibility of causation between the intervention and the AE.

10.1.1. Assessing the severity of an adverse event

If an adverse event occurs, it is evaluated according to the following severity criteria

- Mild: Discomfort but not interfering with daily activities
- Moderate: A participant has some discomfort that limits or affects daily activities.
- Severe: Unable to work or engage in normal daily activities

10.1.2. Evaluating the consequences of adverse events

In the event of an adverse event, the consequences of the adverse event are evaluated according to the following Outcome evaluation criteria

- Recovered/Resolved
- Recovering/Resolving
- Recovered/Resolved with Sequelae
- Not Recovered/Not Resolved
- Fatal
- Unknown

10.2. What to do about adverse events

In the event of an adverse event, the action taken in response to the intervention is evaluated based on the following Action taken evaluation criteria

- Pause
- Permanent suspension
- Not Applicable (Not Applicable)

In addition, any additional actions taken in the event of an adverse event are evaluated against the following Other Action taken evaluation criteria

© 2025. Han MH et al. Published online at http://www.aginganddisease.org/EN/10.14336/AD.2025.0645

- medication given
- Non-drug therapy
- Other
- None

10.3. What to do if a serious adverse event (SAE) occurs

During this study, the principal investigator and staff shall ensure the safety of participants, and in the event of a serious adverse event, take prompt and appropriate action to minimize the adverse event by ensuring that the patient receives the necessary tests and treatment.

In the event of a "serious adverse event" during a clinical trial, each person has the following responsibilities

(1) Investigator Obligations

The investigator shall report any serious adverse events that occur during the clinical trial to the Institutional Review Board and the sponsor in accordance with the relevant regulations of each site and, if necessary, stop part or all of the intervention.

(2) Duties of IRBs

In the event of a serious adverse event, the IRB should take appropriate action, including ordering the discontinuation of part or all of the intervention, if necessary, to the principal investigator.

10.4. Reporting adverse events

- The Principal Investigator will educate investigators and subjects or their guardians about any adverse events that may occur after the intervention and train them to report any phenomena that occur after the intervention.
- For any systemic or clinic-pathologic symptoms that occur during the intervention, the type, date of occurrence, severity, treatment, therapeutic agent, outcome, and causal relationship to the intervention shall be recorded and maintained in the patient case record in accordance with Good Clinical Practice.

10.5. Tracking adverse events

The investigator will observe the adverse event experienced by the subject until the adverse event is closed or the subject is lost to follow-up. Closure of the adverse event means that the subject has returned to baseline or, in the opinion of the investigator, the subject's condition is no longer expected to improve or worsen.

11. Ethical considerations

11.1. Comply with applicable laws and ethical principles

This study is designed in accordance with the ethical principles described in the Declaration of Helsinki and the ICH Harmonized Tripartite Guidelines for Good Clinical Practice and will be conducted and reported in accordance with these principles.

© 2025. Han MH et al. Published online at http://www.aginganddisease.org/EN/10.14336/AD.2025.0645

SUPPLEMENTARY DATA

11.2. Procedures for obtaining human subject consent

Only subjects who meet the inclusion criteria provide written consent on an IRB-approved informed consent form. Informed consent is obtained before any procedures outlined in the study protocol are performed.

11.3. Adherence to the protocol

The researcher will fulfill their obligations to ensure that no protocol violations occur.

11.4. Monitoring studies

Monitoring is conducted to protect the rights and welfare of participants, to ensure that reported study-related data are accurate, complete, and verifiable against supporting documentation, and to ensure that the study is conducted in accordance with the approved protocol, Good Clinical Practice, and applicable local laws and ethical principles. Monitoring of the study will be accomplished through periodic site visits by monitors designated by the multi-center principal investigator in accordance with Good Clinical Practice (GCP), the protocol, and applicable regulations, as well as pre-monitoring by eCRF prior to the visit. During these visits, the monitor will primarily check the original patient records, study files, etc. The monitor will also monitor the progress of the study and discuss any issues with the investigator. The appropriate time for these visits will be determined by the researcher and the monitor. The researcher should make sure that the monitor has access to the patient's original records to verify the data entered in the case notes.

11.5. Changes to the protocol

All changes and additions to the protocol must be approved by the IRB by submitting a written revised protocol. Only changes necessary for the safety of Participants may be implemented prior to IRB approval. If necessary for the safety of Participants, the researcher must take immediate action, even if it means violating the protocol. In such cases, notify the IRB at the lead institution of this action within 10 working days.

11.6. Confidentiality

Keep all subject names confidential and identify subjects during recording and evaluation by the number assigned to them during the study. Inform subjects that all study data will be stored on a computer and will be treated as strictly confidential. The signed informed consent form will be kept by the Principal Investigator. By signing this protocol, the Principal Investigator agrees to properly obtain informed consent from subjects participating in the study and agrees to provide due diligence if requested. The Principal Investigator will maintain a list of subject numbers and subject names so that records can be located at a later date. The consent form and list of subjects must be kept for three years.

11.7. Insurance and liability

© 2025. Han MH et al. Published online at http://www.aginganddisease.org/EN/10.14336/AD.2025.0645

The Sponsor will obtain insurance for the subjects for the entire duration of the study with respect to the risks of the research based on this protocol. In the event of injury or disability resulting from participation in the study, the subject shall promptly notify the treating physician.

11.8. Managing researchers' documents

Data obtained in connection with the conduct of the study shall be stored for three years after the end of the study in accordance with the relevant regulations and then destroyed. The data will be properly and securely stored so that only researchers authorized by the principal investigator are allowed access, and the data will be viewed anonymously for medical purposes only, and the identity of the subjects will be kept confidential if the results are published.

12. References

1. Jorm AF, Dear KB, Burgess NM. Projection of future numbers of dementia cases in Australia with and without prevention. Aust N Z J Psychiatry 2005;39(11-12):959-63.
2. Livingston G, Huntley J, Sommerlad A, et al. Dementia prevention, intervention, and care: 2020 report of the Lancet Commission. Lancet. 2020;396:413-446.
3. Ngandu T, Lehtisalo J, Solomon A, et al. A 2 year multidomain intervention of diet, exercise, cognitive training, and vascular risk monitoring versus control to prevent cognitive decline in at-risk elderly people (FINGER): a randomised controlled trial. Lancet 2015;385(9984):2255-63.
4. Lee KS, Lee Y, Back JH, et al. Effects of a multidomain lifestyle modification on cognitive function in older adults: an eighteen-month community-based cluster randomized controlled trial. Psychother Psychosom 2014;83(5):270-8.
5. Park HK, Jeong JH, Moon SY, et al. South Korean study to prevent cognitive impairment and protect brain health through lifestyle intervention in at-risk elderly people: protocol of a multicenter, randomized controlled feasibility trial. J Clin Neurol 2020;16:292-303.
6. Moon SY, Hong CH, Jeong JH, et al. Facility-based and home-based multidomain interventions including cognitive training, exercise, diet, vascular risk management, and motivation for older adults: a randomized controlled feasibility trial. Aging (Albany NY) 2021;13. doi: 10.18632/aging.203213.
7. Deckers K, van Boxtel MP, Schiepers OJ, et al. Target risk factors for dementia prevention: a systematic review and Delphi consensus study on the evidence from observational studies. Int J Geriatr Psychiatry 2015;30(3):234-46.
8. Chalmers J, MacMahon S, Mancia G, et al. 1999 World Health Organization-International Society of Hypertension Guidelines for the management of hypertension. Guidelines sub-committee of the World Health Organization. Clin Exp Hypertens 1999; 21:1009-1060.

© 2025. Han MH et al. Published online at http://www.aginganddisease.org/EN/10.14336/AD.2025.0645

9. American Diabetes Association. The Expert Committee on the Diagnosis and Classification of Diabetes Mellitus. Report of the expert committee on the diagnosis and classification of diabetes mellitus. Diabetes Care 2018; 41(Suppl 1):S13-S27.
10. National Cholesterol Education Program (NCEP) Expert Panel on Detection, Evaluation, and Treatment of High Blood Cholesterol in Adults (Adult Treatment Panel III). Third Report of the National Cholesterol Education Program (NCEP) Expert Panel on Detection, Evaluation, and Treatment of High Blood Cholesterol in Adults (Adult Treatment Panel III) final report. Circulation 2002;106:3143-421.
11. Pan WH, Yeh WT. How to define obesity? Evidence-based multiple action points for public awareness, screening, and treatment: an extension of Asian-Pacific recommendations. Asia Pac J Clin Nutr 2008;17(3):370–4.
12. Nam GE, Park HS. Perspective on diagnostic criteria for obesity and abdominal obesity in Korean adults. J Obes Metab Syndr. 2018; 27:134-142.
13. Alberti KG, Eckel RH, Grundy SM, et al. Harmonizing the metabolic syndrome: a joint interim statement of the International Diabetes Federation Task Force on Epidemiology and Prevention; National Heart, Lung, and Blood Institute; American Heart Association; World Heart Federation; International Atherosclerosis Society; and International Association for the Study of Obesity. Circulation 2009;120:1640–45.
14. Centers for Disease Control and Prevention (CDC). Cigarette smoking among adults-United States, 1992, and changes in the definition of current cigarette smoking. MMWR Morb Mortal Wkly Rep 1994;43:342-6.
15. World Health Organization. Global recommendations on physical activity for health. Geneva: World Health Organization, 2010.
16. Moon M-Y, Kim B-S, Choi H-R, et al. Effect of Social Participation on Cognitive Function among Aged people in Korea. Korean J Fam Pract 2015;5(3, suppl. 2):708-13
17. Akbaraly TN, Portet F, Fustinoni S, et al. Leisure activities and the risk of dementia in the elderly: results from the Three-City Study. Neurology 2009;73(11):854-61.
18. Chin J, Park J, Yang S, et al. Re-standardization of the Korean-Instrumental Activities of Daily Living (K-IADL): Clinical Usefulness for Various Neurodegenerative Diseases. Dement Neurocognitive Disord 2018;17(1):11-22
19. Han C, Jo SA, Jo I, et al. An adaptation of the Korean Mini-Mental State Examination (K-MMSE) in elderly Koreans: demographic influence and population-based norms (the AGE study). Arch Gerontol Geriatr 2008;47:302-10.
20. Hosking DE, Eramudugolla R, Cherbuin N, et al. MIND not Mediterranean diet related to 12-year incidence of cognitive impairment in an Australian longitudinal cohort study. Alzheimers Dement 2019; 15:581-589.

© 2025. Han MH et al. Published online at http://www.aginganddisease.org/EN/10.14336/AD.2025.0645
